# The contribution of silencer variants to human diseases

**DOI:** 10.1186/s13059-024-03328-1

**Published:** 2024-07-08

**Authors:** Di Huang, Ivan Ovcharenko

**Affiliations:** Intramural Research Program, National Library of Medicine, National Institutes of Health, Bethesda, MD 20892 USA

**Keywords:** Deep learning, Disease-causal single-nucleotide polymorphisms (SNPs), Dual functional regulatory elements, Gene regulation, Silencers

## Abstract

**Background:**

Although disease-causal genetic variants have been found within silencer sequences, we still lack a comprehensive analysis of the association of silencers with diseases. Here, we profiled GWAS variants in 2.8 million candidate silencers across 97 human samples derived from a diverse panel of tissues and developmental time points, using deep learning models.

**Results:**

We show that candidate silencers exhibit strong enrichment in disease-associated variants, and several diseases display a much stronger association with silencer variants than enhancer variants. Close to 52% of candidate silencers cluster, forming silencer-rich loci, and, in the loci of Parkinson’s-disease-hallmark genes TRIM31 and MAL, the associated SNPs densely populate clustered candidate silencers rather than enhancers displaying an overall twofold enrichment in silencers versus enhancers. The disruption of apoptosis in neuronal cells is associated with both schizophrenia and bipolar disorder and can largely be attributed to variants within candidate silencers. Our model permits a mechanistic explanation of causative SNP effects by identifying altered binding of tissue-specific repressors and activators, validated with a 70% of directional concordance using SNP-SELEX. Narrowing the focus of the analysis to individual silencer variants, experimental data confirms the role of the rs62055708 SNP in Parkinson’s disease, rs2535629 in schizophrenia, and rs6207121 in type 1 diabetes.

**Conclusions:**

In summary, our results indicate that advances in deep learning models for the discovery of disease-causal variants within candidate silencers effectively “double” the number of functionally characterized GWAS variants. This provides a basis for explaining mechanisms of action and designing novel diagnostics and therapeutics.

**Supplementary Information:**

The online version contains supplementary material available at 10.1186/s13059-024-03328-1.

## Introduction

A common but often elusive goal of biological investigations is to uncover the genetic basis of disease phenotypes [1, 2]. This is challenging due to the inherent complexity of human genetics. Although genome-wide association studies (GWASs) offer valuable genetic insights into diseases and disorders, they struggle to pinpoint causative variants due to linkage disequilibrium among genetic variants. Notably, a significant majority of GWAS variants, exceeding 90%, occur within noncoding genomic regions [3]. To accurately map disease-causal variants, it is vital to characterize the function of non-coding regions. Up to now, the investigations have primarily focused on well-characterized non-coding regulatory elements including enhancers, promoters, and insulators [4–8]. These studies consistently underscore the impact of regulatory elements on disease.

Evidence has also indicated pathological roles of silencers, however. For instance, a rare silencer variant disrupts the binding of NR2F1 and affects the expression of *GATA2* in neurons leading to hereditary congenital facial paresis type 1 [9]. Another variant deactivates a silencer in breast cells, causing the overexpression of *ESR1* and *RMND1* in breast cancer [10].

Despite these and a few similar discoveries, silencers have been underexplored in genetic and genomic research, in general, primarily due to the difficulties in systematically profiling these elements across the whole genome [11]. Recent advancements in massively parallel reporter assays (MPRAs) and computational analysis tools have allowed genome-wide mapping of silencers [12–16], opening doors to in-depth investigations into the association of silencers with diseases and phenotypic traits in humans.

Understanding the regulatory effects of non-coding variants is a key challenge in genetic research, essential to discovering molecular causes of diseases [17, 18]. Here, we apply a deep learning framework to a diverse collection of 97 biological samples (biosamples), building a deep learning model in each biosample to detect biosample-specific candidate silencers. Our results demonstrate that candidate silencers are enriched in disease-associated regulatory single-nucleotide polymorphisms (SNPs), but their disease-association profiles differ from those of enhancers. We demonstrate how silencer modeling can be used to predict the regulatory impact of variants within candidate silencers and to identify disease-causal variants.

## Results

### Genome-wide silencer landscape in 97 cell types

**Fig. 1:**
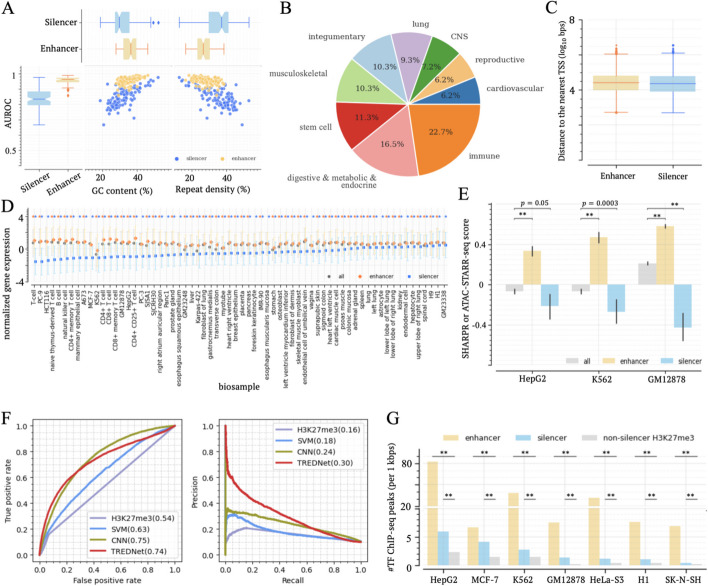
Profiling candidate silencers across 97 biosamples from diverse origins. **A** Classification performance (AUROCs) of TREDNet models for silencers and enhancers in analyzed biosamples. AUROCs exhibit a correlation with GC contents and repeat densities of training sequence sets. Each dot represents a set of enhancers or silencers. **B** Distribution of 97 biosamples across cell types. **C** Distance of candidate silencers and enhancers to their nearest TSSs. **D** Expression of genes proximal to candidate silencers and enhancers. Markers and their flanking lines represent the medians and standard deviations of gene expression levels. Blue and orange asterisks on the top represent the significantly low and high expression levels, respectively, compared to all genes ($$p<0.05$$). **E** MPRA scores of candidate silencers and enhancers in three biosamples. **F** Performance of the TREDNet model on MPRA silencers. **G** Densities of TF ChIP-seq peaks within candidate silencers and enhancers across biosamples. $$**:p<{10}^{-10}$$

We trained two-phase deep learning TREDNet models [19] to predict enhancers and silencers, building a multi-class classifier for each of the biosamples collected by the ENCODE project (see “Methods”). Albeit lower than the 0.96 enhancer area under receiver operating characteristic curve (AUROC), the accuracy of silencer prediction was on par with our prior models (0.84 AUROC) [14] and was significantly better than AUROC = 0.77 of our prior support vector machine (SVM) models [20]. While these SVM models employ DNA sequences and gene expression profiles for silencer prediction, TREDNet models are DNA sequence-based, and thus can be readily extended to additional biosamples. These AUROC values exhibit a positive correlation with GC content levels and a negative correlation with repeat density (Fig. 1A). This partially explains lower classification performance on silencers than on enhancers since enhancer sequences (defined as DNase-seq and H3K27ac ChIP-seq peaks) generally feature higher GC content and lower repeat density than silencers (defined as DNase-seq and H3K27me3 ChIP-seq peaks that lack overlap with H3K27ac peaks, see “Methods”). With the trained TREDNet models, we identified enhancers and silencers in each biosample, and conservatively selected 97 biosamples with over 5000 candidate enhancers and silencers in them for further investigation (Additional file 1: Table S1). These biosamples encompass a diverse array of human cell types, including but not limited to 22 immune biosamples (20 including blood cells, spleen, and thymus), 16 digestive, metabolic, and endocrine biosamples, and 7 biosamples from the central nervous system (Fig. 1B and Additional file 1: Table S1). Among them, 20 (20%) biosamples are from cancer cell lines.

We identified a total of 2.8 million candidate silencers and 5.8 million enhancers (see “Data and tools in Methods”), collectively spanning approximately 37.6% of the human genome. In cancer biosamples, 10% exhibit a higher count of silencers than enhancers, a proportion notably lower than the 16.5% of all examined biosamples (binomial test $p=0.007$, Additional file 2: Fig. S1). This finding is consistent with gene overexpression in cancer cells [21], which might be due to silencer loss or deactivation in cancer. On average, 57.7% of candidate silencers and 42.9% of candidate enhancers are located within intergenic regions (binomial test $p<{10}^{-10}$, Additional file 2: Fig. S2). Nonetheless, silencers and enhancers exhibit comparable distances to their nearest transcriptional start site (TSS), with approximately half of them residing within 26 kb of their nearest TSS (Fig. 1C).

Examining the evolutionary conservation, we noticed that an average of 8.7% of candidate silencer sequences and 10.6% of enhancer sequences overlap genomic regions conserved across 30 primate species (Siepel et al. 2005), significantly exceeding the 5.7% expectation stemming from the whole human genome (Student’s *t* test $p<{10}^{-10}$, Additional file 2: Fig. S3). This underscores the negative selective pressure imposed on functional genomic regions to preserve their biological function (Siepel et al. 2005), but also reflects a rapid turnaround of regulatory elements in vertebrates [22]. In 63.6% (14/22) of immune biosamples, candidate silencers are more conserved than enhancers, significantly higher than the 34% of all biosamples (binomial test $p=3\times {10}^{-9}$). This finding highlights the significance of candidate silencers in an immunological context. For example, the loci of *PCDH* genes, which are highly conserved in vertebrates [23] and play an important role in epithelial barrier formation and repair, display the enrichment in candidate silencers, but not enhancers, in immune biosamples (Additional file 2: Fig. S4). The trend is also evident in the highly conserved loci of *HOXA* and *HOXD* clusters (Additional file 2: Fig. S4), developmentally essential genes associated with embryonic development [24].

### Functional evaluation of silencer predictions

To assess the impact of candidate silencers, we initially analyzed the expression of genes located near these elements across 66 biosamples with available gene expression profiles from the ENCODE project (see Additional file 1: Supplementary Notes) since genes associated with active silencers are likely to be lowly expressed. Across all examined biosamples, genes neighboring candidate silencers exhibit significantly lower expression than all assayed genes ($p<0.05$, Fig. 1D). Similarly, genes targeted by candidate silencers, as determined by Hi-C chromatin loops [25], consistently display low expression across all tested biosamples ($p<0.05$, Additional file 2: Fig. S5).

Furthermore, we directly evaluated the activity of candidate silencers by utilizing the experimental results from MPRA platforms designed to measure the silencing or activating impact of genomic regions. In K562 and HepG2 biosamples, candidate silencers frequently exhibit negative scores reported by the Sharpr-MPRAs [26]. These scores are significantly lower than those observed in enhancers and all tested regions (Wilcoxon rank-sum test $\text{p}\le 0.05$, Fig. 1E), supporting the active silencing function of candidate silencers. Similarly, in GM12878, significant negative ATAC-STARR-seq scores, which represent “silent” genomic sequences [27], are enriched among candidate silencers ($\textit{p}=4\times10^{-16}$ vs all tested sequences, Fig. 1E).

Additionally, we compiled 7701 K562 silencers from two independent MPRA studies based on ReSU [13] and STARR-seq [12]. Of them, 541 overlap with K562 predicted silencers, which represents a significant enrichment compared to the DNase-seq peaks randomly selected from alternative biosamples and H3K27me3 ChIP-seq peaks not predicted as silencers in K562 cells (binomial test $p<{10}^{-10}$, Additional file 2: Fig. S6A). Similarly, in HepG2 cells, predicted silencers are significantly enriched with silencers detected by the ReSU MPRA [13] ($p<{10}^{-10}$, Additional file 2: Fig. S6B).

Moreover, we validated the TREDNet silencer model on an independent experimental dataset of MPRA silencers. After excluding MPRA silencers overlapping sequences used for training the TREDNet model, we had 6999 K562 MPRA silencers remaining for validation. On this subset of MPRA silencers, the TREDNet model demonstrates a classification performance of AUROC = 0.74 and AUPRC = 0.30 with the 1:9 ratio of positive to control samples. It shows a marginal improvement over our prior CNN classifier [14] and significantly outperforms our prior SVM model [20] and general H3K27me3 signal profiles (Fig. 1G). Furthermore, the TREDNet silencer model can effectively distinguish both H3K27me3 and non-H3K27me3 MPRA silencers from control sequences (Additional file 2: Fig. S7). These results reaffirm that the TREDNet silencer model can identify active silencers with respectable accuracy.

To further investigate whether candidate silencers actively suppress gene expression as opposed to being genomic regions of repressed chromatin, we analyzed the abundance of transcription factor binding sites (TFBSs) and chromatin contacts, under the assumption that repressed chromatin regions host significantly fewer TFBSs and chromatin contacts than active enhancer and silencer regions. In each of tested biosample with ChIP-seq data for more than 50 TFs available from the ENCODE project, candidate silencers contain, on average, 3.5 times as many TF ChIP-seq peaks as H3K27me3 ChIP-seq peaks lacking candidate silencers (Wilcoxon rank-sum test $p<{10}^{-10}$, Fig. 1E). Additionally, the density of Hi-C chromatin contacts within predicted silences is 1.5 times greater than the corresponding density within H3K27me3 ChIP-seq peaks lacking candidate silencers (binomial test $p<0.05$, Additional file 2: Fig. S8).

Overall, these results support that the TREDNet predicted silencers predominantly act as active silencers and not simply heterochromatic regions of the genome. Therefore, we refer to them as candidate silencers.

### Candidate silencers are associated with development

**Fig. 2:**
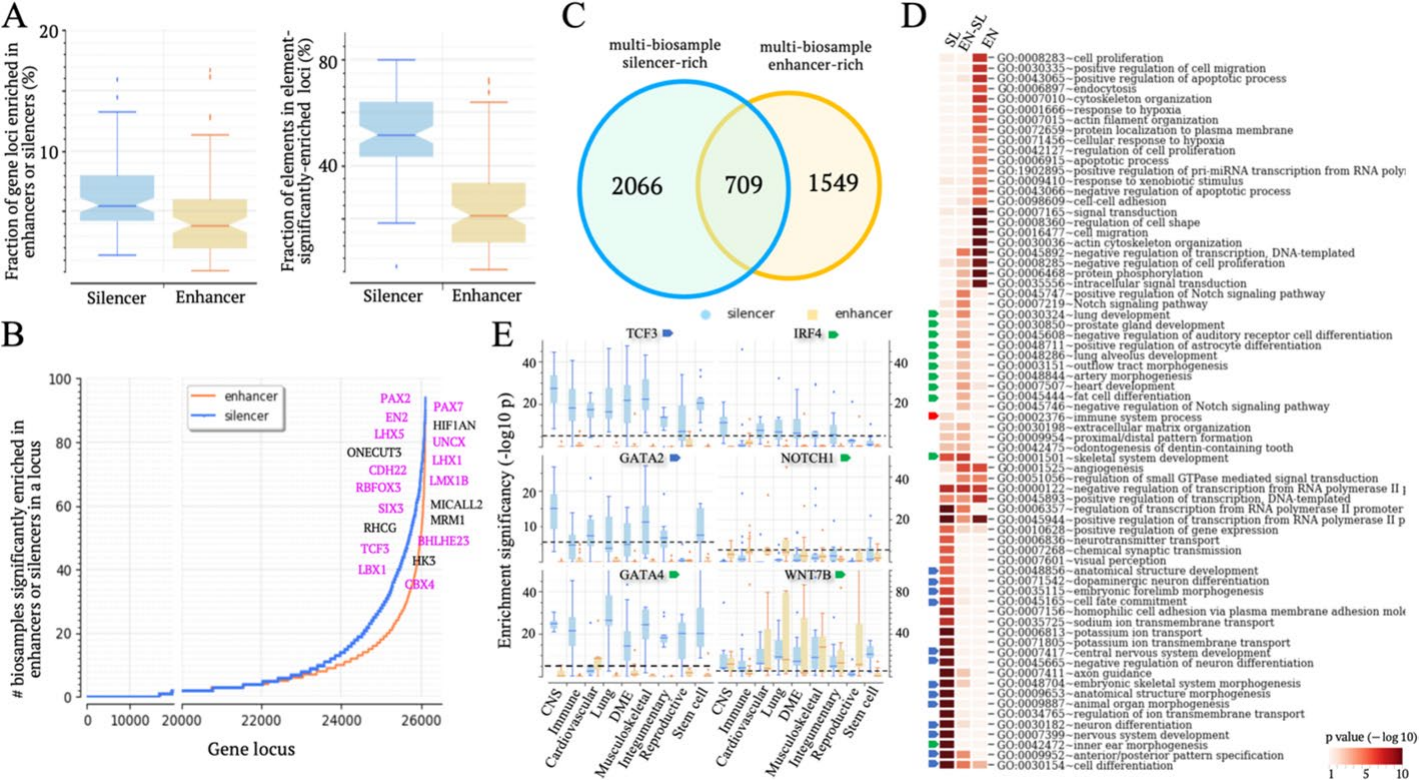
Candidate silencers are significantly associated with development and immunity. **A** Fractions of silencer-rich or enhancer-rich gene loci are shown in the left panel. Proportions of candidate silencers located within silencer-rich loci and enhancers located within enhancer-rich loci are shown in the right panel. **B** Frequency of gene loci exhibiting silencer-richment (blue line) or enhancer-richment (orange line) across biosamples. Top-frequency silencer-rich gene loci are listed, among which developmental loci are highlighted in pink. **C** Numbers of multi-biosample silencer-rich and enhancer-rich gene loci. Notably, 709 gene loci are both multi-biosample silencer-rich and enhancer-rich. **D** Heatmap illustrating biological processes significantly associated with different gene sets. SL and EN represent multi-biosample silencer-rich and enhancer-rich gene loci, respectively. ENSL represents the intersection of SL and EN sets. Biological processes in embryonic and central nervous system (CNS) development are indicated by blue arrows, while immunity regulation and tissue-specific development are by red and green arrows, respectively. **E** Enrichment of candidate silencers and enhancers in six gene loci. The dashed lines represent the threshold ($\text{p}=1.9\times {10}^{-6}$) for significant enrichment. DME represents digestive, metabolic, and endocrine biosamples. The upper and lower whisker edges in these boxplots represent approximately 25 and 75% quartiles of the presented data

To evaluate biological functions associated with candidate silencers, we turned to their nearby genes. Genomic proximity to a specific class of genes, although not comprehensive enough to capture long-range chromatin interactions, is commonly used to examine biological functions of regulatory elements [28]. We defined the locus of a gene as its gene body along with the entire intergenic areas between this gene and its nearest neighbors. On average, 6.3% of gene loci are enriched in candidate silencers with a significance of $p<{10}^{-5}$ compared to the whole genome (referred to as “silencer-rich gene loci”). This percentage is substantially higher than the 4.7% of gene loci showing enhancer enrichment (Student’s *t* test $p=0.0007$, Fig. 2A). Across biosamples, silencer-rich loci harbor 51.7% of all silencers, notably higher than the 25.8% of enhancers found in enhancer-rich loci (Student’s *t* test $p=2\times {10}^{-22}$, Fig. 2A), suggesting a pronounced trend of candidate silencer accumulation in specific gene loci.

Among the gene loci displaying the highest frequency of candidate silencer enrichment across biosamples are *PAX2*, *PAX7*, *EN2*, *HIF1AN*, and *LHX5* (Fig. 2B). All of them are known as essential for development. Gene loci significantly enriched in candidate silencers in over-9 biosamples from different groups are denoted as multi-biosample silencer-rich gene loci. In total, there are 2775 such gene loci (Fig. 2C). These genes are associated with fundamental developmental processes and neurological system development (DAVID $p<{10}^{-6}$, indicated by blue arrows, Fig. 2D) [29]. Additionally, these gene loci are notably associated with immune system regulation ($p=0.002$). For example, the loci of cell-differentiation regulators *TCF3* and *GATA2* show elevated densities of candidate silencers in 90 and 54.6% of examined biosamples, respectively. The *IRF4* locus, crucial for the immune system, displays a significant enrichment in candidate silencers in 71.4% of CNS cells.

On the other hand, multi-biosample enhancer-rich gene loci are involved in housekeeping biological processes such as signal transduction, cell–cell adhesion, and protein phosphorylation ($p<{10}^{-3}$, Fig. 2D). Furthermore, there are a total of 709 gene loci that are both multi-biosample enhancer-rich and silencer-rich, thus termed as multi-biosample enhancer-silencer-rich (Fig. 2C). These genes often take part in tissue-specific developmental processes (indicated by green arrows in Fig. 2D). For example, the locus of *GATA4*, a key factor in heart, pancreatic and hepatic development, is enhancer-rich in cardiovascular biosamples but silencer-rich in 50% of other biosamples (Fig. 2E). The locus of *WNT7B*, encoding a signal protein crucial for tissue development, is silencer-rich in 79.4% of biosamples and enhancer-rich in 53.6% of them. In summary, candidate silencers are preferentially distributed in the proximity of the genes controlling fundamental and tissue-specific developmental processes, significantly associated with the regulation of these genes. These results suggest that the regulation of developmental genes is often tightly orchestrated with an array of enhancer and silencer elements establishing a complex multi-cellular regulatory profile.

### Silencer-to-enhancer transitions are a hallmark of cellular differentiations

Functional transitions between enhancers and silencers across biological contexts are pivotal in the precise and expeditious regulation of developmental processes [14, 30]. A substantial portion of candidate silencers and enhancers reported here have dual functions. Specifically, 55% of candidate silencers and 42% of enhancers are dual functional regulatory elements (DFREs), acting as enhancers in certain biosamples but as silencers in others (Additional file 2: Fig. S9).

**Fig. 3:**
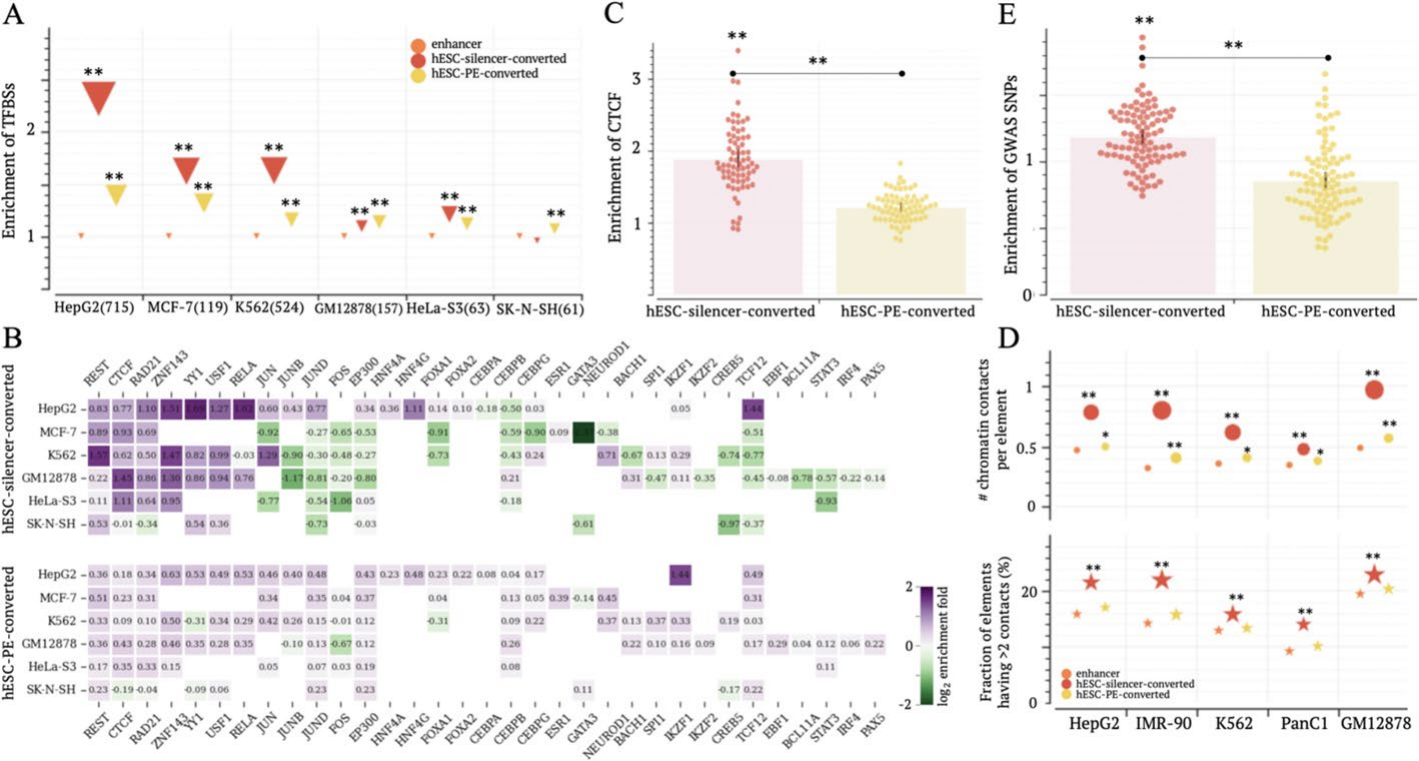
hESC-silencer-converted enhancers anchor chromatin loops. **A** Enrichment of ChIP-seq TFBSs in hESC-silencer-converted and hESC-PE-converted enhancers in comparison to all enhancers. The numbers in parentheses are the number of TFs examined in this study. **B** Enrichment of TFBSs for individual TFs. The blank cells indicate an absence of TF ChIP-seq data. **C** Enrichment of CTCF ChIP-seq TFBSs across 69 biosamples. **D** Numbers of chromatin contacts per element (the top panel) and the fractions of elements having > 2 contacts (the bottom panel) in hESC-silencer-converted enhancers. Additional results are presented in Additional file 2: Fig. S10. **E** Enrichment of GWAS SNPs within hESC-silencer-converted and hESC-PE-converted enhancers in comparison to all enhancers across biosamples. $$*:p<0.01$$ and $$**:p<{10}^{-10}$$

Moreover, 68% of candidate silencers of H1 human embryonic stem cells (H1-hESCs) are converted to enhancers in partially or fully differentiated biosamples examined in this study. These enhancers contain significantly more TFBSs than other enhancers in five out of six tested biosamples ($p<{10}^{-10}$, Fig. 3A). This significance remains evident even when compared to the enhancers that are converted from H1-hESC poised enhancers (PEs, defined as H3K4me1 ChIP-seq peaks carrying no H3K27ac modification signals in H1-hESCs). For example, in K562 cells, each hESC-silencer-converted enhancer harbors an average of 58 TF ChIP-seq peaks, significantly more than the 35 found in all K562 enhancers and the 42 in K562 hESC-PE-converted enhancers ($p<{10}^{-10}$). Moreover, compared to other enhancers (including PE-converted enhancers), hESC-silencer-converted enhancers are enriched in TF ChIP-seq peaks of dual functional TFs like YY1 and chromatin organizers such as CTCF, RAD21, and ZNF143. On the other hand, these enhancers lack TF ChIP-seq peaks of cell-specific transcriptional activators like CEBPB in HepG2 cells, ESR1 and NEUROD1 in MCF-7 cells, BACH1 and EBF1 in K562 cells, and IRF4 and BCL11A in GM12878 cells (Fig. 3B). Furthermore, in 94% (65/69) of the biosamples for which CTCF ChIP-seq data are available in the ENCODE project (Additional file 1: Table S2), hESC-silencer-converted enhancers show significantly higher densities of CTCF ChIP-seq peaks compared to all enhancers, including hESC-PE-converted ones (Fig. 3C), with an average enrichment fold of 1.8. The pronounced enrichment in TF ChIP-seq peaks, particularly for CTCF, hints that hESC-silencer-converted enhancers frequently serve as anchors for chromatin loops, a crucial aspect in chromatin organization [31].

To further verify this interpretation, we analyzed chromatin contacts of enhancers (as defined by Hi-C data, see “Methods”). In the biosamples where over 20% of enhancers have reported Hi-C contacts, hESC-silencer-converted enhancers display the highest density of Hi-C contacts ($p<{10}^{-10}$, Fig. 3D). Importantly, they hold at-least-3 chromatin contacts more frequently than other enhancers ($p<{10}^{-10}$). In HepG2 cells, 21.5% of hESC-silencer-converted enhancers have at-least-3 chromatin contacts, significantly higher than the 15.8% of all enhancers and the 17.1% of hESC-PE-converted enhancers ($p<{10}^{-10}$, Fig. 3D). These trends persist in biosamples where fewer than 20% of enhancers have Hi-C contacts, although statistical significance diminishes possibly due to limited detection of chromatin contacts (Additional file 2: Fig. S10). These results reaffirm that hESC-silencer-converted enhancers often serve as anchors for chromatin loops.

To further assess the functional significance of hESC-silencer-converted enhancers, we utilized the single-nucleotide polymorphisms (SNPs) annotated in GWASs. We downloaded GWAS SNPs documented in the National Human Genome Research Institute (NHGRI) catalog [32] and in the UK Biobank release 2 cohort [33]. After the inclusion of the SNPs in tight linkage disequilibrium (LD ${\text{r}}^{2}>0.8$) with GWAS SNPs, a total of 2.2 million GWAS SNPs were compiled, which are associated with 1116 distinct traits (Additional file 2: Fig. S11, see “Methods”). HESC-silencer-converted enhancers exhibit a significant increase ($p<0.01$) in the density of GWAS SNPs compared to all enhancers in 75% (69/92) of differentiated biosamples (Fig. 3E). This increase remains significant even when compared to H1-hESC-PE converted enhancers ($p<{10}^{-10}$). In 73% (67/92) of differentiated biosamples, GWAS SNP densities in hESC-silencer-converted enhancers are significantly higher than those in hESC-PE-converted enhancers. These findings support the functional importance of these enhancers, partially due to their role as anchors for chromatin loops.

### GWAS studies suggest a critical role of candidate silencers in neurological and autoimmune disorders

**Fig. 4:**
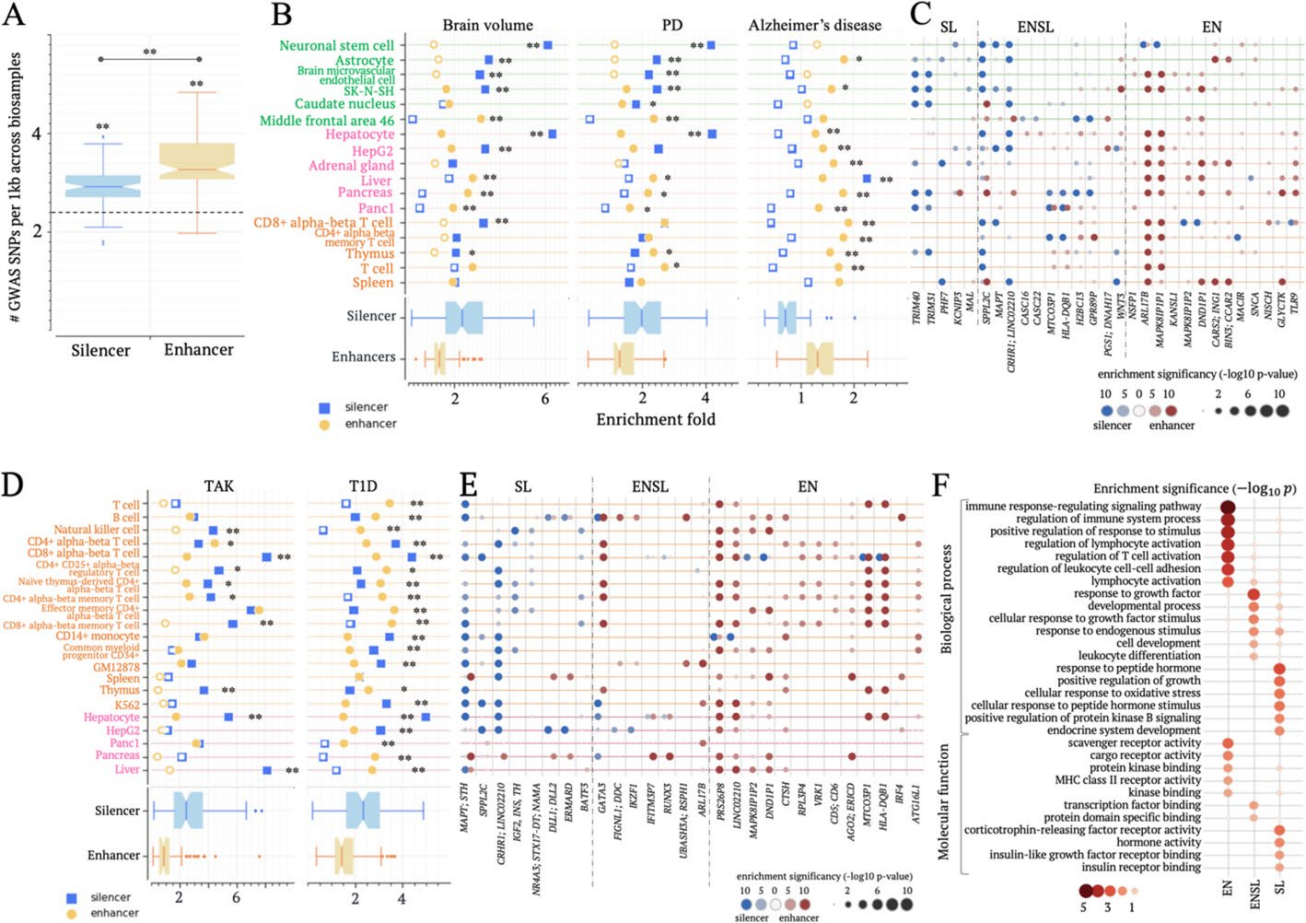
Candidate silencers exhibit the enrichment for GWAS SNPs. **A** Numbers of GWAS SNPs per 1 kb in candidate silencers and enhancers across biosamples. The dashed line represents the number of GWAS SNPs per 1 kb in the whole genome. **B** Enrichments of SNPs associated with brain volume, PD, and Alzheimer’s disease within candidate silencers and enhancers across biosamples. Asterisks indicate the significant difference between candidate silencers and enhancers. **C** Enrichments of PD-associated SNPs within candidate silencers and enhancers in individual gene loci. Only gene loci having significant enrichments are included here. **D** Enrichments of SNPs associated with TAK and T1D within candidate silencers and enhancers. In **B** and **D**, enrichment folds are estimated in comparison to the whole genome. Significant enrichments are denoted by solid markers ($$p<{10}^{-5}$$). The results on other autoimmune diseases are presented in Additional file 2: Fig. S16. **E** Enrichment of T1D-associated SNPs within candidate silencers and enhancers in individual gene loci. In **C** and **E**, gene loci are clustered based on the enrichment profiles of associated SNPs. SL/EN represents the gene loci where the associated SNPs are enriched exclusively in candidate silencers/enhancers, while ENSL denotes the gene loci where the associated SNPs are enriched in both candidate silencers and enhancers. **F** Functional analysis results for T1D-associated gene clusters defined in **E**. $$**:p<{10}^{-5}$$ and $$*:p<0.01$$

We further utilized GWAS SNPs to assess the phenotypic impact of all candidate silencers. On average, candidate enhancers and silencers in examined biosamples harbor 3.4 and 3.0 NHGRI GWAS SNPs per 1 kb, respectively. Both values are significantly higher than the 2.4 whole genome GWAS SNPs density (Student’s *t* test $p<{10}^{-20}$, Fig. 4A).

Similarly, candidate silencers exhibit significant enrichment in expression quantitative trait loci (eQTLs) obtained from the GTEx project [34] compared to the whole genome across 28 out of 40 examined biosamples (Additional file 2: Fig. S12A and Supplementary Notes). Additionally, candidate silencer eQTLs achieve significance levels akin to enhancer eQTLs across these biosamples (Additional file 2: Fig. S12B). Silencer eQTLs are, however, more tissue-specific than enhancer eQTLs in 90% of examined biosamples (36/40; $p<0.05$, Additional file 2: Fig. S12C). Furthermore, we explored the distribution of GWAS SNPs deposited to the ClinVar archive [35]. Candidate silencers host 1.47 ClinVar SNPs per 1 kb. This density exceeds 1.29 ClinVar SNP per 1 kb within enhancers, with both densities significantly surpassing the expected 0.76 ClinVar SNP per 1 kb baseline from the whole genome ($p<{10}^{-5},$ Additional file 2: Fig. S13A). We also examined the distribution of cancer somatic variants compiled in the ICGC database [36]. These cancer variants show significant enrichment within candidate silencers in the matched biosamples for seven out of eight examined cancers (Additional file 2: Fig. S13B). For example, the density of myeloid cancer variants in K562 candidate silencers is 1.3 times that expected from the whole genome baseline. Taken together, these findings suggest an observable phenotypic impact of candidate silencers.

Notably, GWAS SNPs associated with different traits have varying enrichment levels in candidate silencers and enhancers across biosamples (Additional file 1: Table S3). For example, SNPs associated with Alzheimer’s disease are predominantly located in CNS and immune system enhancers ($p<{10}^{-10}$ versus the whole genome as marked by a solid symbol, Fig. 4B). In contrast, SNPs associated with Parkinson’s disease (PD) are preferentially located in candidate silencers in five out of six brain biosamples $(p<{10}^{-5}$ versus the whole genome and enhancer counterparts) and within enhancers in immune biosamples (Fig. 4B). SNPs associated with brain volume traits, such as intracranial, hippocampal, thalamus, and subiculum volume, are notably biased toward candidate silencers in four out of six brain biosamples ($p<{10}^{-5}$ versus the whole genome and enhancer counterparts, Fig. 4B).

To further dissect the genetic basis of PD, we evaluated the enrichment levels of associated SNPs within candidate silencers and enhancers in each gene locus (see “Methods”). In the locus of *TLR9*, a gene known for its involvement in the degeneration of dopamine neurons in PD [37], PD-associated SNPs mainly cluster in brain enhancers (Fig. 4C). In contrast, the *TRIM31* locus, responsible for metal ion binding, harbors a total of 104 PD-associated SNPs, a number significantly higher than the genome-wide average ($p<{10}^{-30}$). Of these SNPs, 18 are located within SK-N-SH candidate silencers, which is notably higher than 8 SNPs as expected in the TRIM31 locus. Interestingly, no PD-associated SNPs are found within the *TRIM31* SK-N-SH candidate enhancers. This pronounced bias to brain candidate silencers is also observed in the loci of *MAL* and *MAPT*, both associated with neurogenesis (Fig. 4C). These findings consistently underscore the significant role of brain candidate silencers in PD, particularly in relation to metal ion binding and neurogenesis, two factors closely linked to PD (Additional file 2: Fig. S14) [38, 39].

We also analyzed the genetic mechanisms underlying differences in brain volume. The SNPs associated with brain volume are enriched within candidate enhancers in the loci of *CTBP2* and *ZRANB1* in brain biosamples and *KANSL1* in immune biosamples. These SNPs are enriched in candidate silencers in the locus of *DMRAT2* in brain biosamples (Additional file 2: Fig. S15). *DMRTA2* is key in controlling the cell cycle during neuronal differentiation. Its dysregulation may lead to severe microcephaly [40], suggesting the crucial contribution of brain candidate silencers to brain volume measurement and, more broadly, the development of the brain.

Similarly, across autoimmune disorders, candidate enhancers and silencers in immune and endocrine biosamples show varying enrichments for GWAS SNPs. For example, while enriched within both candidate enhancers and silencers ($p<{10}^{-5}$ vs the whole genome), SNPs associated with rheumatoid and system lupus erythematosus (SLE) exhibit a distinct predilection for immune enhancers but for endocrine candidate silencers (silencers vs enhancers: $p<{10}^{-5}$, Additional file 2: Fig. S16). On the other hand, osteoarthritis-associated SNPs are biased toward candidate silencers over enhancers in immune system biosamples (silencers vs enhancers: 2.3 vs 2.0 of the average enrichment, binomial test $p={10}^{-20}$). Takayasu’s arteritis (TAK) associated SNPs are preferentially situated within candidate silencers in immune system biosamples (silencers vs enhancers: 3.9 vs 2.4 of the average enrichment, $p={10}^{-11}$, Fig. 4D). Especially, in the MICA locus, TAK-associated SNPs are clustered within candidate silencers, rather than enhancers, in immune system biosamples (Additional file 2: Fig. S17). Given that the upregulation of the MIC family in blood vessels contributes to the stimulation of natural killer cells in TAK [41], it is plausible that the deactivation of candidate silencers in immune system biosamples could underlie the etiology of TAK.

Interestingly, SNPs associated with type 1 diabetes (T1D), a T-cell-mediated autoimmune disease that attacks pancreatic $\beta$ cells [42], are notably prevalent within both candidate silencers and enhancers across immune system and endocrine biosamples (Fig. 4D). However, these SNPs display varying preferences for candidate silencers and enhancers within individual gene loci (Fig. 4E). Gene loci enriched with T1D-associated enhancer SNPs govern immune processes and/or the activity of receptors (Fig. 4F). Instances include *IRF4*, *CD5*, *CD6*, and *CTSH*. In contrast, T1D-associated silencer SNPs congregate conspicuously within the loci of *INS*, *IGF2*, and several other genes responsive to or producing hormones, notably insulin. Overexpression of *IGF2* renders pancreas islets susceptible to immune onslaught, thereby potentially serving as a key biomarker of T1D pathogenesis [43]. Our finding proposes that silencer variants in *IGF2* locus may contribute to T1D risk and identify a handful of specific silencer SNPs, which could be targeted in follow-up clinical and biochemical studies.

In short, candidate silencers and enhances, thought governing distinct functions, jointly drive crucial biological progress in complex diseases, as exemplified here by PD, T1D, and TAK. However, silencers’ contributions to these diseases are not identical to those of enhancers.

### Candidate silencers underlie the genetic difference between bipolar disorder and schizophrenia

**Fig. 5:**
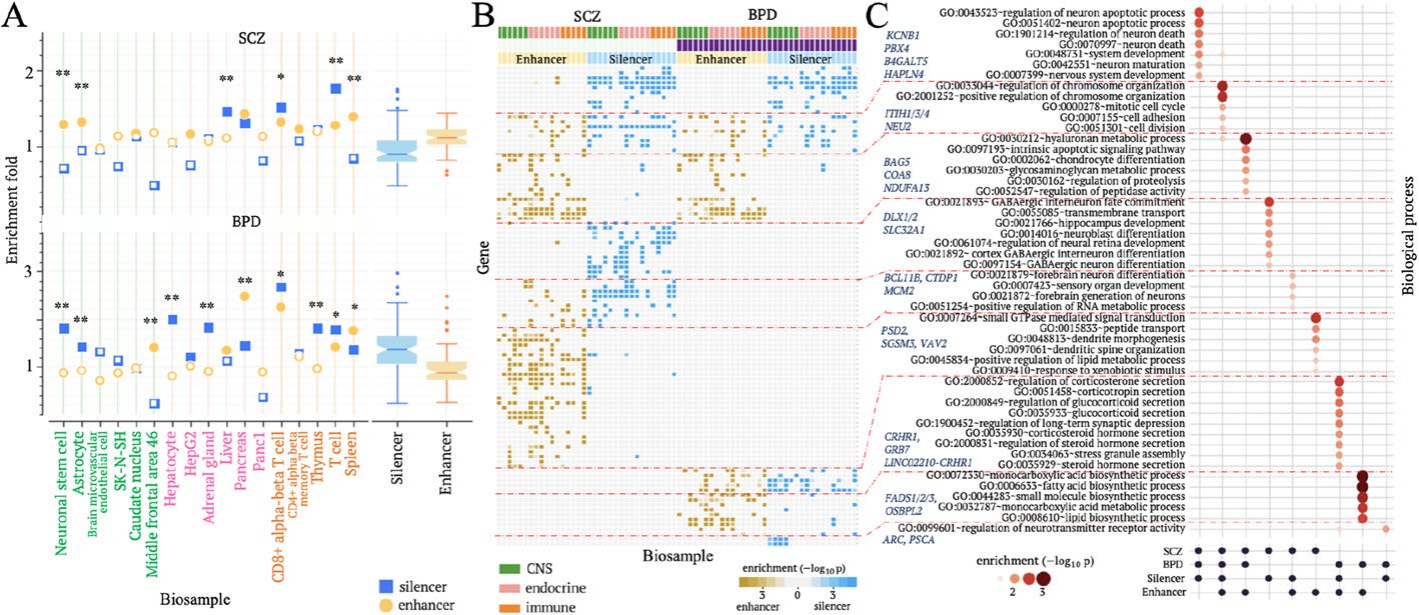
Candidate silencers distinguish SCZ from BPD. **A** Enrichments of SNPs associated with BPD and SCZ within candidate silencers and enhancers across biosamples. Enrichment folds are estimated in comparison to the whole genome. Significant enrichments are denoted by solid markers ($$p<{10}^{-5}$$). Asterisks above markers indicate the significant difference between candidate silencers and enhancers. **B** Heatmap depicting the clusters of gene loci associated with SCZ and/or BPD, based on the enrichment profiles of associated SNPs within candidate silencers and enhancers. Each column represents the enrichment of SCZ or BPD associated SNPs within candidate silencers or enhancers in a biosample. The biosamples presented here are the same as those in **A**. **C** Functional analysis of gene clusters defined in **B**. $$*:p<0.001$$ and $$**:p<{10}^{-5}$$

To demonstrate the application of candidate silencer (and enhancer) profiles in a disease genetic study, we investigated regulatory mechanisms of bipolar disorder (BPD) and schizophrenia (SCZ). These two neurodevelopmental disorders, with a genetic correlation of over 0.6 based on common SNPs [44], share substantial overlap in both genetics and symptomology. The identification of shared and distinct genetic components between SCZ and BPD constitutes a fundamental stride toward deciphering the mechanisms of these diseases and formulating targeted therapeutic interventions [45]. To address this objective, we utilized candidate silencer and enhancer profiles in brain, immune system, and endocrine biosamples, given the notable involvement of endocrine and immune systems in these disorders [46, 47]. SNPs associated with SCZ and/or BPD are enriched in candidate silencers and enhancers across endocrine and immune biosamples (Fig. 5A). SCZ-associated SNPs are enriched in brain candidate enhancers, while BPD-associated SNPs are preferentially distributed within brain candidate silencers ($p<0.001$ vs the whole genome, Fig. 5A).

To further elucidate genetic factors contributing to SCZ and BPD, we analyzed the distribution of their associated SNPs in each gene locus. Both SCZ- and BPD-associated SNPs display enrichment within enhancers in the loci of genes responsible for housekeeping biological activities like intrinsic apoptosis and hyaluronan metabolic process ($p<0.01$, Fig. 5B). In contrast, these SNPs are commonly found within candidate silencers in the loci of brain-specific genes, particularly those controlling the apoptosis of neuronal cells and brain development. For example, the locus of *KCNB1*, a key gene in the voltage-gated potassium channel crucial for neuron development and apoptosis [48], harbors 38 SCZ-associated SNPs and 21 BDP-associated SNPs. These numbers significantly exceed the expected by chance from the whole genome ($p<{10}^{-22}$). Among 38 SCZ-associated SNPs in the *KCNB1* locus, 10 (21.1%) are located within astrocyte candidate silencers, a notable preference as compared to the mere 1.2% of all SCZ-associated SNPs found in astrocyte candidate enhancers (binomial test $p={10}^{-11}$). Similarly, in the *KCNB1* locus, 8 (38.1%) of the BDP-associated SNPs are located within astrocyte candidate silencers, significantly higher than the 2.9% observed for all BPD-associated SNPs across the whole genome (binomial test $p={10}^{-7}$). The significant association of SCZ and BDP with neuron development and apoptosis, consistent with the previous findings [49, 50], emphasizes the crucial role of silencer variants in the susceptibility to BPD and SCZ (Fig. 5B).

Interestingly, despite an insignificant enrichment in brain candidate silencers on a genome-wide level, SCZ-associated SNPs exhibit a distinct enrichment within candidate silencers in the loci of genes controlling the differentiation of GABAergic interneuron cells and hippocampus development (Fig. 5C). Aberrant activity of GABAergic neurons has been reported as a key site of SCZ pathology [51]. Our finding proposes that this anomaly is greatly attributable to the variants in CNS candidate silencers, thereby offering a lead for further biological examinations.

On the other hand, BPD-associated SNPs are enriched within both candidate silencers and enhancers in the loci of genes regulating corticosterone secretion and long-term synaptic depression. These two biological processes have been observed to be dysregulated in BPD patients [52, 53]. In summary, analyzing candidate silencer and enhancer profiles alongside GWAS results can unveil the biological mechanisms that differentiate diseases with similar origins, as demonstrated by the analysis of BPD and SCZ here.

### Disease-associated silencer variants alter binding affinities of TFs

**Fig. 6:**
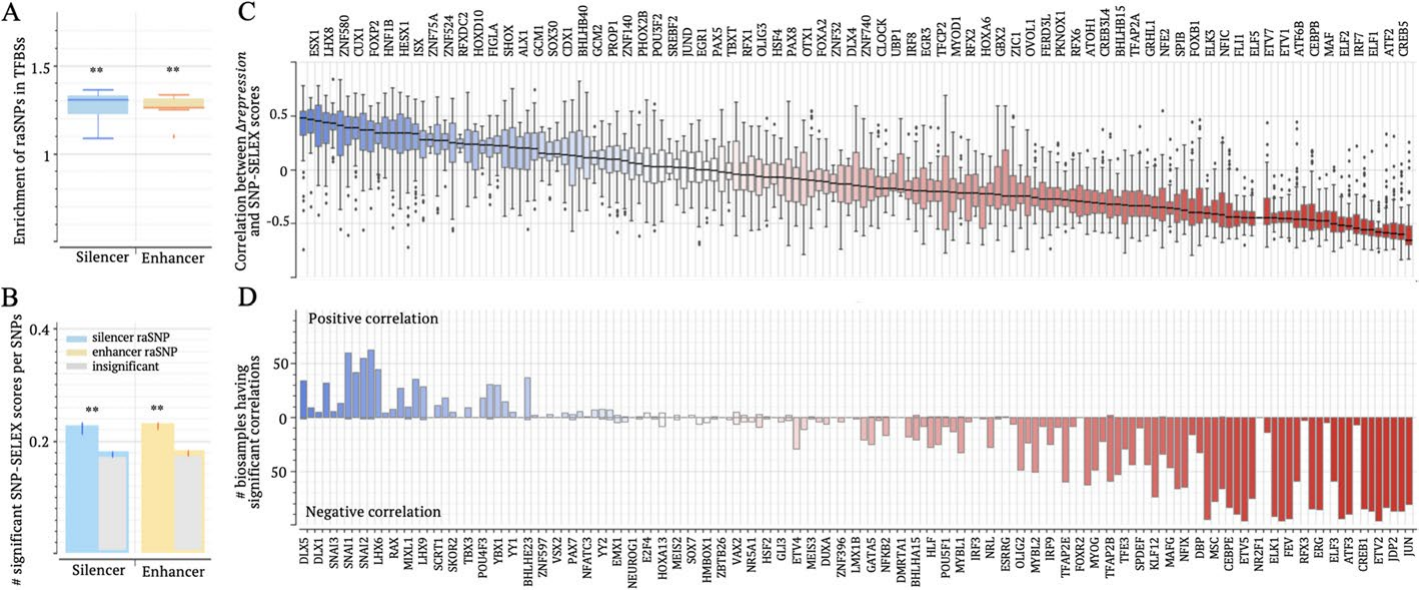
$$\Delta repression$$ significantly correlates with SNP-SELEX scores. **A** Enrichments of raSNPs in TFBSs (as defined in TF ChIP-seq peaks) in seven biosamples. **B** Enrichments of significant SNP-SELEX scores among raSNPs. **C** Correlations between $$\Delta repression$$ and SNP-SELEX scores for each TF across biosamples. **D** Numbers of biosamples exhibiting significantly positive (above zero line) and negative (below zeros line) correlations between $$\Delta repression$$ and SNP-SELEX scores for each TF. TF names are displayed along the top *x*-axis in **C** and the bottom *x*-axis in **D** combined. $$**:p<{10}^{-10}$$

Our investigation next proceeded to the analysis of individual SNPs, aiming to identify disease-causal or trait-determining non-coding variants among GWAS SNPs [14, 17]. We quantified the impact of SNPs on gene regulation by comparing prediction scores from a trained TREDNet model between SNP alleles, denoted as $\Delta repression$ (see “Methods”). A positive $\Delta repression$ suggests a decrease in repressive activity due to a given SNP. SNPs with a significant $\Delta repression$ are marked as regulatory-activity-alternating SNPs (raSNPs, see “Methods”). RaSNPs are more frequently found in TF ChIP-seq peaks than common SNPs across seven biosamples (binomial test $p<{10}^{-10}$, Fig. 6A). To prevent possible bias of raSNPs toward specific TFs, all seven biosamples examined in this study include ChIP-seq peaks for more than 50 TFs (see “Methods”). In HepG2, a candidate-silencer raSNP coincides with an average of 2.1 TF ChIP-seq peaks, which is 1.22 times the average for all common SNPs within candidate silencers ($p<{10}^{-10}$, Fig. 6A). Similarly, in enhancers, TF ChIP-seq peak densities at raSNPs are 1.33 times those at all common SNPs ($p<{10}^{-10}$).

We then evaluated allele-specific TF-binding affinities of raSNPs. Allele-specific TF-binding affinities of SNPs were measured in a multiplex protein-DNA binding assay, known as systematic evolution of ligands by exponential enrichment (SNP-SELEX), for 270 TFs in the HepG2 cell line [54]. Significant SNP-SELEX scores, which indicate substantial difference in binding affinities between SNP alleles, frequently occur among raSNPs across all examined biosamples. The occurrence rates of significant SNP-SELEX scores at raSNPs are over 1.26 times those at SNPs with insignificant-$\Delta repression$ scores, within either candidate silencers or enhancers (binomial test $p<{10}^{-10}$, Fig. 6B, see “Methods”). These high occurrence frequencies, together with the enrichment of raSNPs in TF ChIP-seq peaks, highlight the significant possibility of raSNPs altering TF binding affinities.

Importantly, $\Delta repression$ scores positively correlate with SNP-SELEX scores of transcription repressors. For the repressors FOXP1 and SNAI1/2 [55], these positive correlations are significant (linear regression $p<0.05$) in over-50 biosamples (Figs. 6C, D). Of the raSNPs having significant SNP-SELEX scores for FOXP1, 69% show the directional concordance between $\Delta repression$ and SNP-SELEX scores (Additional file 2: Fig. S18). This concordance rate is over 65% for SNAI1/2. In contrast, $\Delta repression$ scores negatively correlate with SNP-SELEX scores of transcription activators. For prominent activators like JUN, CREB5, ELF1/2, CEBPE, NFE2, and SPIB, these negative correlations remain significant in over-50 biosamples. On average, the directional discordance rates between $\Delta repression$ and SNP-SELEX scores for these TFs is 67%. As positive SNP-SELEX scores indicate a reduction in binding affinity from wild-type to mutant alleles, these substantial positive or negative correlations (and directional concordance or discordance rates) underscore the effectiveness of $\Delta repression$ scores in capturing the impact of SNPs on binding affinity for both transcriptional repressors and activators. Additionally, bifunctional TFs like YY2 and PAX5, which act as both activators and repressors, rarely present a significant $\Delta repression$-SNP-SELEX correlation in examined biosamples (Figs. 6C, 6D and Additional file 2: Fig. S18).

For example, the SNP rs11065189, associated with SCZ but not BPD, is situated within a candidate silencer in brain microvascular endothelial cells. The substitution from G to A results in a significant decrease in the binding affinity of the transcriptional activators MAF, MAFG, and NRL. These measurements align with $\Delta repression= -0.49$, the highest magnitude within its 5 kb vicinity (Additional file 2: Fig. S19).

In summary, these three lines of TF-binding-based evidence consistently substantiate the functional potency of raSNPs and the accuracy of $\Delta repression$ scores in evaluating the influence of SNPs on TF-binding affinity.

### The role of silencer SNPs in PD, SCZ, and other neurological diseases

To directly evaluate the relationship between $\Delta \text{repression}$ scores and raSNPs, we resorted to the outcomes of MPRA experiments that assess allele-specific impacts of SNPs on gene regulation. Although these MPRA platforms were not specifically tailored for silencer SNPs, they provide valuable insights. For example, in SuRE MPRA experiments conducted in K562 cells [56], 19,237 SNPs were reported to significantly alter regulatory activity, known as reporter assay QTLs (raQTLs). These raQTLs are extremely enriched in K562 enhancers, consistent with previous findings [56]. Nevertheless, we also observed a significant enrichment of raQTLs in candidate silencers and K562 MPRA silencers compared to the whole genome and H3K27me3 ChIP-seq peaks not classified as silencers (binomial test $p<{10}^{-10}$), although these silencer enrichment levels are notably lower than that in enhancers ($p<{10}^{-10}$, Additional file 2: Fig. S20A), as expected from the nature of the experimental data. This enrichment further supports the active state of K562 candidate silencers. In addition, $\Delta \text{repression}$ s are positively correlated with raQTL scores, irrespective of whether these raQTLs are in silencers or enhancers (Additional file 2: Fig. S20B). Taken together, MPRA scores by which the difference in regulatory influence between SNP alleles are quantified, though not specifically designed for silencer SNPs, can be used to examine the performance of $\Delta \text{repression}$ s in prioritizing disease-risk SNPs within candidate silencers.

**Fig. 7:**
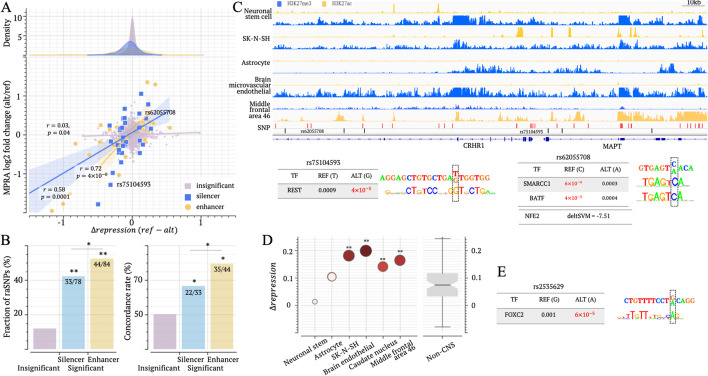
Brain raSNPs have a strong regulatory impact. **A** Correlation between $$\Delta repression$$ and dementia MPRA scores in a neuronal stem cell. The top panel illustrates $$\Delta repression$$ score distributions for different SNP groups. The bottom panel plots $$\Delta repression$$ and MPRA scores of SNPs. SNP groups here are insignificant-MPRA SNPs, significant-MPRA silencer, and enhancer SNPs. The analysis results in other brain biosamples are presented in Additional file 2: Fig. S21. **B** Fractions of raSNPs (the left panel) and directional concordance between $$\Delta repression$$ and MPRA scores (the right panel) across SNP groups. In the left panel, the numbers of all examined SNPs and raSNPs among these SNPs are listed in the bars. **C** Epigenetic profile of silencer SNPs associated with PD in MAPT locus. TF binding motif mapping results on example SNPs are also presented. In the track of “SNP,” black and red bars represent tag PD SNPs and their LD SNPs, respectively. **D** $$\Delta repression$$ scores of SCZ-associated SNP rs2533629 in brain biosamples. **E** Analysis of TF binding motif mapping at rs2533629. $$*:p<0.05$$ and $$**:p<{10}^{-5}$$

To directly evaluate the regulatory impacts of raSNPs in candidate silencers in brain biosamples, we utilized their MPRA scores for dementia GWAS SNPs [57]. Positive/negative MPRA scores directly indicate increased/decreased regulatory activation due to sequence variants. In neuronal stem cells, SNPs with significant MPRA scores have a plateau distribution of $\Delta repression$ scores, unlike insignificant-MPRA-score SNPs (Fig. 7A). More precisely, 52.4 and 42.3% of significant-MPRA-score enhancer and silencer SNPs were labeled as a raSNP, significantly higher than the 12.8% of all insignificant-MPRA-score SNPs ($p<{10}^{-10}$, Fig. 7B) and the 18.9% of insignificant-MPRA-score within enhancers and silencers ($p<{10}^{-5}$).

Notably, $\Delta repression$ scores in neuronal stem cells positively correlate with MPRA scores. This positive correlation remains significant regardless of MRPA scores and SNP locations ($p=0.04, r=0.03$ among insignificant-MRPA-score SNPs; $p=0.0001, r=0.58$ among significant-MPRA-score silencer and $p=4\times {10}^{-8}, r=0.72$ among significant-MPRA-score enhancer SNPs, Fig. 7A).

Among significant-MPRA-score silencer SNPs, $\Delta repression$ scores are directionally concordant to the corresponding MPRA scores in over two-thirds of instances (Fig. 7B). This concordance rate is significantly higher than the 50% for insignificant-MPRA-score SNPs (binomial test $p=0.04$). The robust correlation between $\Delta repression$ scores and MPRA scores is also evident in other brain biosamples. In these biosamples, the concordance rate is 67.5% among raSNPs ($p={10}^{-9}$ vs 51.0% of insignificant-MPRA-score SNPs, Additional file 2: Fig. S21). Altogether, these findings strongly support the high accuracy of $\Delta repression$ scores in gauging the regulatory effects of variants, at least in brain biosamples.

Focusing on specific SNPs, we started with the SNP rs62055708, which is associated with PD and many other neurological traits, including autism, bipolar disorder, brain volume measurement, and intelligence. It is a SNP located within candidate silencers in most brain biosamples except the middle frontal area (Fig. 7C). The C to A change at this SNP has $\Delta repression=0.20$ in neuronal stem cells, aligning with an MPRA-score of 0.42. Also, this SNP corresponds to reduced significance in binding motif mapping for transcriptional repressors SMARCC1 (the allele C vs A: $p=6\times {10}^{-6}$ vs $0.0003$) and BATF ($p=4\times {10}^{-5}$ vs $0.0004$, Fig. 7C, see “Methods”) [58, 59]. Additionally, as predicted by SNP-SELEX deltaSVM [54], the change from the allele C to A at this SNP gains a binding site for NFE2, a transcriptional activator as discussed above (Fig. 6). Another PD-associated SNP is rs75104593. Consistent MPRA-score =  − 1.28 and $\Delta repression=-0.32$ in neuronal stem cells suggest that the substitution at this SNP (from T to G) boosts the repressive effect, which could be supported by the increased significance of binding motif mapping for REST, a well-known repressor TF (Fig. 7C). It is worth noting that both REST and NFE2 are widely recognized as PD-associated factors [60, 61], further strengthening the connection between these two raSNPs and PD.

At a SCZ-associated rs2535629, a substitution from G to A has been experimentally confirmed to increase the binding affinity of CTCF in a ChIP‐Allele‐Specific‐qPCR assay and diminish the suppressive impact in a dual‐luciferase reporter gene assay [62]. This SNP is a raSNP located within candidate silencers in four out of six examined brain biosamples. The $\Delta repression$ scores in brain biosamples are significantly higher than in non-brain biosamples (Student’s* t* test $p={10}^{-21}$, Fig. 7D). TF-motif-mapping analysis also shows increased binding affinity of FOXC2 due to the G to A change at this SNP (Fig. 7E). FOXC2 is a transcription activator contributing to gene overexpression in various cancers, like glioblastoma [63]. This finding provides an additional mechanistic clue to understanding the potential role of rs2535629 in the development of SCZ. The strong agreement of $\Delta repression$ with MPRA scores and TF binding affinity prediction underscores the high accuracy of $\Delta repression$ scores in assessing the regulatory impact of genetic variants.

### T1D and other autoimmune diseases are linked to variants in candidate silencers

To assess $\Delta repression$ scores in immune biosamples, we compared them with MPRA scores measured in lymphoblastoid cell lines from two independent studies, i.e., the multiplex MPRAs, denoted as mMPRA below [64] and the variant-based MPRAs, referred to as vMPRA [65].

**Fig. 8:**
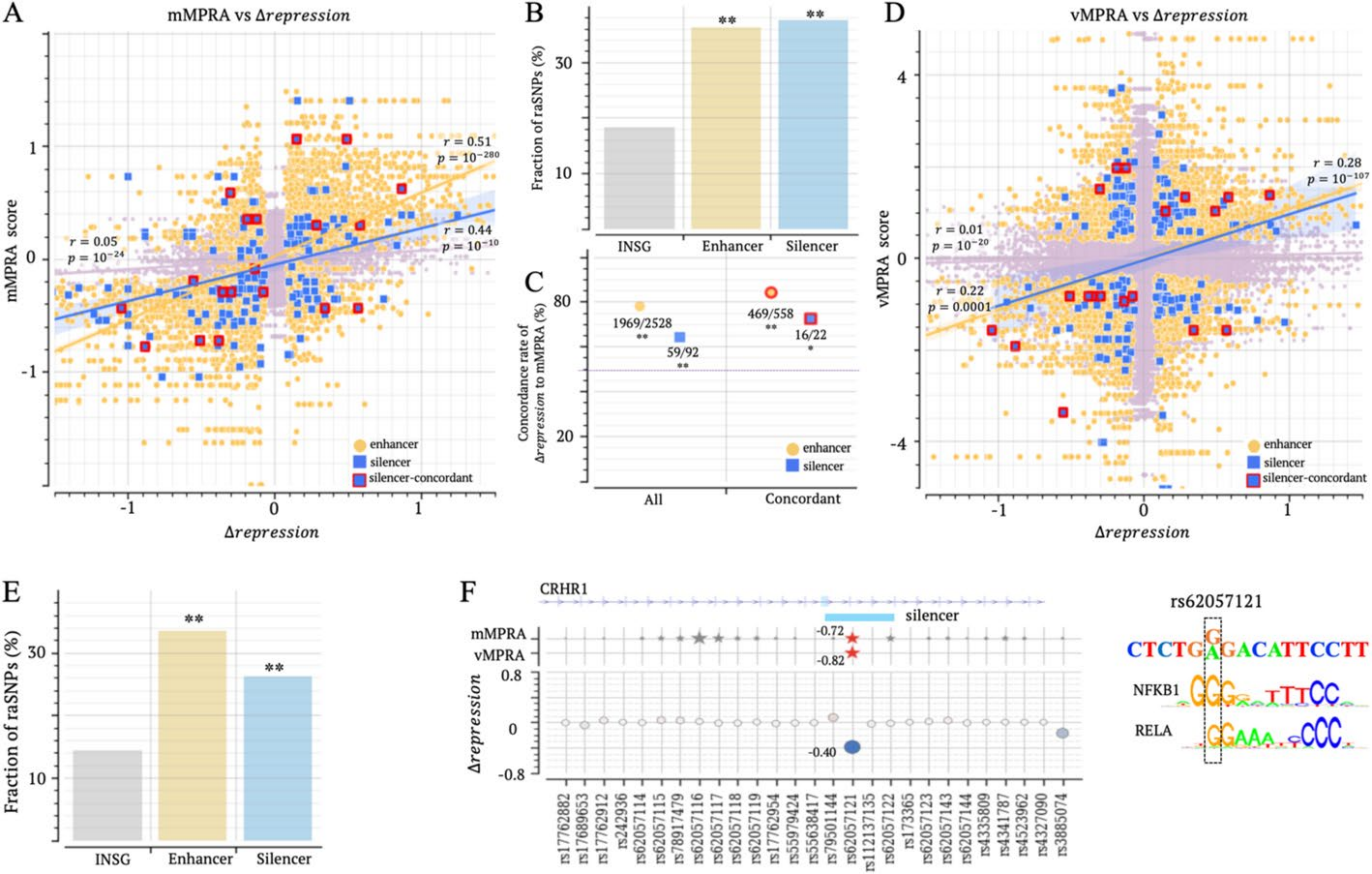
Immune raSNPs within candidate silencers have a strong regulatory impact. **A** Correlations between $$\Delta repression$$ and mMPRA scores in SNP groups. Silencer-concordant represents the silencer SNPs where significant mMPRA and vMPRA scores directionally align. **B** Fractions of raSNPs among insignificant-mMPRA, significant-mMPRA silencer/enhancer SNPs. **C** Concordance rate between $$\Delta repression$$ and mMPRA score across SNP groups. “All” represents all significant-mMPRA SNPs in candidate silencers or enhancers. “Concordant” is as denoted in **A**. Numbers alongside each marker indicate the count of SNPs where $$\Delta repression$$ and mMPRA scores are directionally concordant, as well as the total number of SNPs considered. The dashed line represents the expectation for randomly shuffling $$\Delta repression$$ scores. **D** Correlations between $$\Delta repression$$ and vMPRA scores. **E** Fractions of raSNPs among insignificant-vMPRA, significant-vMPRA silencer/enhancer SNPs. **F** $$\Delta repression$$, mMPRA, vMPRA scores on the T1D-associated rs62057121 and its neighboring SNPs. In the top panel, red/gray stars indicate significant/insignificant mMPRA or vMPRA scores, respectively. All significant scores are listed next to the corresponding markers. In addition, the TF binding motif analysis on this SNP is presented. $$*:p<0.05$$ and $$**:p<{10}^{-8}$$

SNPs with significant mMPRA scores show a higher magnitude of $\Delta repression$ than insignificant-mMPRA-score SNPs (Fig. 8A and Additional file 2: Fig. S22). Specifically, 37 and 36% of significant-mMPRA-score SNPs in candidate silencer and enhancer are raSNPs in immune biosamples, significantly surpassing the 19% of insignificant-mMPRA-score SNPs ($p<{10}^{-10}$, Fig. 8B). Notably, $\Delta repression$ scores in immune cells are significantly positively correlated with mMPRA scores across different SNP sets ($p<{10}^{-10}$ across insignificant-mMPRA-score and candidate silencer/enhancer significant-mMPRA-score SNPs).

Furthermore, 64.1% of raSNPs in candidate silencers have a $\Delta repression$ score directionally concordant to their mMPRA scores, significantly exceeding the 49.4% as expected from randomly shuffling $\Delta repression$ scores, as well as the 51.4% of SNPs with insignificant MPRA scores ($p<0.01$, Fig. 8C). This concordance rate further increases to 72.3% among the SNPs where mMPRA and vMPRA scores directionally align, although these increases are not significant most likely due to the shrinking size of the analyzed SNP set (Fig. 8C). Similar trends are mirrored among enhancer SNPs. Additionally, $\Delta repression$ scores exhibit significant positive correlations with vMPRAs in immune biosamples ($p<0.0005$, Figs. 8D and Additional file 2: S23). For example, 33 and 26% of significant-vMPRA-score SNPs in candidate silencers and enhancers are raSNPs in immune biosamples, significantly surpassing the 14% of insignificant-vMPRA-score SNPs ($p<{10}^{10}$, Fig. 8E). These significant correlations and high concordance rates are in the line with the observations on dementia MPRAs (Fig. 7), generalizing the high validity of $\Delta repression$ scores in evaluating regulatory effects of variants across different biosample groups.

For example, rs6207121, a SNP associated with T1D, exhibits significant scores in mMPRA and vMPRA. This SNP, with $\Delta repression= -0.4$, is detected as a raSNP within a candidate silencer in CD4 + alpha–beta T cells, holding the highest magnitude within its 4 kb vicinity. This $\Delta repression$ score directionally aligns with the corresponding mMPRA and vMPRA scores (Fig. 8F). Moreover, the analysis of binding motif mappings suggests that this variant potentially disrupts a binding site for NFKB1, a key TF known for dual repressive and activating functions in the immune system [66] and in the development of T1D [7].

Another example is the rs242561 SNP, which has been linked to a range of immune and neurological disorders, including T1D, BPD, and Parkinson’s disease. This SNP is predicted as a raSNP in both immune and brain biosamples. The significantly negative $\Delta repression$ scores in brain biosamples correlate with the negative dementia MPRA score (Additional file 2: Fig. S24). Interestingly, this SNP is located within a DFRE, acting as a silencer in immune biosamples but an enhancer in CNS biosamples, likely by recruiting different TFs in immune cells and in neurons.

## Discussion

Here, we report 2.8 million candidate silencers in 97 human biosamples representing diverse origins, collectively spanning 19.4% of the human genome. More than half of candidate silencers (55%) are DFRE, acting as enhancers in alternative biosamples, which evidences the widespread presence of DFREs. Furthermore, the majority (67%) of hESC candidate silencers function as DFREs, which could still increase after additional human biosamples are explored. In differentiated cells, the hESC-silencer-converted enhancers exhibit a notable enrichment in TFBSs of CTCF, RAD21, and ZNF143, as well as in chromatin contacts, suggesting they frequently act as anchors for chromatin contacts.

This study demonstrates the vital role of candidate silencers in complex diseases with a strong genetic basis. This new perspective goes beyond GWAS, uncovering how individual disease-associated genes are regulated during pathogenesis. For example, SCZ and BPD have been linked through GWAS to the dysregulation of neuronal differentiation and apoptosis. Our analysis shows that this dysregulation may primarily stem from variants within brain candidate silencers. Moreover, the disruption of the GABAergic interneuron has been reported as a key cause in SCZ [67]. Our analysis further underpins that the variants within brain candidate silencers could be responsible for this disruption. Similarly, in the gene loci of *INS* and *IGF2*, T1D-associated SNPs are greatly concentrated within candidate silencers, implying the pivotal roles that candidate silencers play in regulating these genes in the immune system. Silencer variants thereby greatly account for the dysregulation of these two genes in the context of T1D [42]. Collectively, silencers represent fundamental components underlying the development of many complex diseases. The profiles of silencers (along with enhancers) can facilitate the unraveling of the genetic basis of these diseases.

It is important to note that this study is centered around silencers, with enhancers serving as a reference point. The goal is to underscore the significance of silencers in disease research, rather than to provide an exhaustive genetic portrait of diseases. Genetic components of diseases that go beyond these elements are not within the scope of this study. For example, we do not delve into *LILR* genes, which host TAK-associated variants in their promoters [41]. Evidently, a comprehensive understanding of a polygenic disease requires the exploration of diverse regulatory elements, along with protein-coding variants, which is the motivation of this study.

We further extended the analysis to the level of individual genetic variants. High correlations with the experimental results from MPRA and SNP-SELEX studies validate the accuracy of $\Delta repression$ scores in predicting the regulatory impact of SNPs across different biosamples. RaSNPs, the SNPs having a significant $\Delta repression$ score, frequently hold significant MPRA scores and SNP-SELEX scores, confirming the substantial impact of these variants on disease susceptibility. Prioritizing disease-causal SNPs is the initial step to reveal molecular mechanisms underlining polygenic diseases. Delineating the cascading effects of these SNPs, such as how they alter TF binding affinity, chromatin organization, and gene expression, represents the subsequent challenge. It is noteworthy that, although we present experimental and computational results of TF binding affinities of raSNPs here, this issue will remain incompletely addressed until experimental profiling of TF binding expands to many more TFs and spans additional cell types across multiple developmental time points. For example, as demonstrated here, experimental results from SNP-SELEX assays are restricted to a small proportion of SNPs, possibly due to their cell specificity [54].

Here, silencer identification primarily relies on H3K27me3 ChIP-seq peaks. While this histone mark is a well-characterized and widely accepted proxy of repressive regulatory influence, our candidate silencer profiles might be incomplete due to the existence of non-H3K27me3 silencers [12, 13]. The strong association of candidate silencers with developmental genes, particularly those active during embryonic stages, aligns with the established role of H3K27me3 in developmental processes [68]. This association may also hint at a possible bias toward H3K27me3 among candidate silencers. Currently, the detection of non-H3K27me3 silencers is limited to few cell types [13, 15, 69] and/or confined to certain genomic regions [70, 71], which largely hampers the investigation of these silencers. Furthermore, although MPRA results and gene expression analysis have shown that candidate silencers suppress gene transcription in several biosamples, additional experimental examinations on candidate silencers across more biosamples and/or using different biotechnological platforms (such as CRISPR) will be beneficial for future silencer investigations. Despite these constraints, our analysis underscores the significance of silencers in controlling key biological processes and highlights their profound influence on disease susceptibility.

## Conclusions

This study examined silencers and the contribution of silencer variants to human diseases. We developed a series of deep learning models to identify silencers across a wide range of human biosamples and demonstrated that the identified silencers are significantly enriched in disease-associated variants. Predicted silencer-disrupting variants are well aligned with the MPRA experimental validation. Furthermore, we report that the developed silencer models can be used for profiling the genetic etiology of complex diseases. For example, the disruption of apoptosis associated with SCZ and BPD can largely be attributed to variants in brain silencers. While silencers have been largely overlooked in gene regulation studies, our research highlights the important role of silencer variants in diseases and underscores the need for more studies focusing on silencer-based gene regulation to achieve a comprehensive understanding of polygenic diseases. Moreover, the silencer datasets and deep learning models generated in this study are likely to serve as a valuable resource for future investigations.

## Methods

### Identification of candidate silencers

We trained the TREDNet model, a two-phase deep learning model [19] to predict enhancers and silencers. We downloaded DNase-seq peaks, H3K27ac and H3K27me3 ChIP-seq peaks (“narrow peak”) for 111 biosamples from ENCODE project (https://www.encodeproject. org/, Additional file 1: Table S1). Enhancer training sequences were defined as the DNase-seq peaks overlapping H3K27ac ChIP-seq peaks but not H3K27me3 peaks in the central 400 bp. Silencer training sequences were defined as the DNase-seq peaks overlapping H3K27me3 peaks but not H3K27ac peaks in the central 400 bp as well as the H3K27me3 peaks not overlapping H3K27ac peaks. To accommodate this multi-label classification task, the output layer of TREDNet models consists of three nodes with the activation function of “softmax,” representing silencer, enhancer, and control samples, respectively. The cost function used here is “categorical cross entropy.” We held out chromosomes 7 and 8 for testing. All other autosomes were used for building the classification model [19]. Consequently, testing sequences, having no overlap with training sequences, provide an unbiased computational evaluation on the performance of the TREDNet models.

For silencer prediction, 1-kb-long input sequences were evaluated by silencer prediction scores. The cutoff for labeling silencers (say ${t}_{s}$) was set as a false positive rate (FPR) of 0.1 in test samples, with control to positive samples in the ratio of 9:1. DNase-seq peaks or H3K27me3 ChIP-seq peaks that have a silencer score greater than ${t}_{s}$ were predicted as silencers. Similarly, the cutoff for labeling enhancers (say ${t}_{e}$) was set as a false positive rate (FPR) of 0.1 in test samples, again with control to positive samples in the ratio of 9:1. DNase-seq peaks that have an enhancer score greater than ${t}_{e}$ were predicted as an enhancer. The sequences marked as both enhancers and silencers were considered as “uncertain,” which account for less than 1% of silencers or enhancers in all tested biosamples and were excluded from further analysis. To this end, 97 biosamples have over-5000 candidate enhancers and over-5000 candidate silencers, which were investigated in this study.

Each candidate enhancer/silencer is 1 kbp long. A candidate silencer in a biosample was considered as a DFRE if it overlaps with an enhancer in another biosample by over-200 bp. Similarly, an enhancer was considered as a DFRE when it overlaps with a candidate silencer in another biosample by over-200 bp.

### GWAS SNP enrichment in individual gene loci

We assess the significance of GWAS SNPs associated with a disease ($i$) in a gene locus ($j$), ${p}_{ij}$, in comparison to the whole genome using the binomial test. The gene loci having a ${p}_{ij}<{10}^{-8}$ are regarded as associated with the disease $i$. Similarly, in a disease-associated locus (say $j$), the enrichment of given GWAS SNPs within silencers, ${p}_{ij}^{s}$, is assessed by using the binomial test. That is,

1 $${p}_{ij}^{s}={\sum }_{m=k}^{N}\left(\begin{array}{c}N\\ m\end{array}\right){{\pi }_{0}}^{m}{\left(1-{\pi }_{0}\right)}^{N-m},$$

where ${\pi }_{0}$ is the ratio of the locus length to the whole genome. $N$ and $k$ are the total number of given GWAS SNPs within the candidate silencers and the number of given GWAS SNPs within the candidate silencers in the locus $j$. The enrichment of given GWAS SNPs in candidate enhancers in the locus $j$ is evaluated by replacing $N$ and $k$ in Eq. (1) with the number of given GWAS SNPs within the enhancers in the whole genome and in the locus $j$, respectively.

$$\Delta {\varvec{r}}{\varvec{e}}{\varvec{p}}{\varvec{r}}{\varvec{e}}{\varvec{s}}{\varvec{s}}{\varvec{i}}{\varvec{o}}{\varvec{n}}$$

To evaluate the regulatory impact of a variant with the wild type (wt) and mutant allele (mu), we input the 1-kb-long sequences centering at this variant to a trained TREDNet model. We then obtained the silencer and enhancer prediction scores for all alleles. The false positive rates of silencer prediction scores (denoted as ${FPR}^{s}$) are evaluated based on test samples with the ratio of control to positive samples = 9:1. Similarly, the false positive rates corresponding to enhancer prediction scores (represented by ${FPR}^{e}$) are evaluated based on test samples. The regulatory alteration between these alleles is then estimated as

$$\Delta repression=\left({\text{log}}_{10}{FPR}_{mu}^{s}-{\text{log}}_{10}{FPR}_{wt}^{s}\right)-\left({\text{log}}_{10}{FPR}_{wt}^{e}-{\text{log}}_{10}{FPR}_{mu}^{e}\right).$$

A positive $\Delta repression$ indicates a decrease in the repressive impact due to the mutation.

In a biosample, we evaluated the significance $p$ value of a $\Delta repression$ score by comparing with $\Delta repression$ scores on all common SNPs documented in dbSNP as of 2017 [72]. A $\Delta repression$ score is regarded as significant if $p<0.05$ among all common SNPs. A SNP is marked as raSNP if the corresponding $\Delta repression$ score is significant. When analyzing the correlation between $\Delta repression$ and MPRA scores (Figs. 7 and 8), SNPs are considered as silencer SNPs either when they are located within a candidate silencer or when they overlap with a H3K27me3 ChIP-seq peak and have ${FPR}_{wt}^{s}<0.05$. Similarly, SNPs are considered as enhancer SNPs either when they are located within a candidate enhancer or when they overlap with a H3K27ac ChIP-seq peak and have ${FPR}_{wt}^{e}<0.05$.

### Data and tools

We downloaded GWAS SNPs curated in the National Human Genome Research Institute (NHGRI) catalog [32] and in UK Biobank release 2 cohort [33] in 2022. All the GWAS SNPs associated with the same trait, according to their Experimental Factor Ontology ID [73], were merged into one SNP set. We extended trait-associated SNP sets by including the SNPs in tight linkage disequilibrium (LD ${\text{r}}^{2}>0.8$) to GWAS SNPs based on the EUR population in 1000 Genomes Project. We used the webtool SNiPA (https://www.snipa.org/snipa3/) [74] to identify LD SNPs in hg19 and then mapped them to hg38. To this end, we retrieved a total of 2.2 million GWAS SNPs, which are associated with 2212 distinct traits. Among these traits, 1166 traits are linked to more than 80 SNPs and thus used in our investigation.

Hi-C chromatin contacts were detected from the study by Salameh et al. [25] and downloaded from http://3dgenome.fsm.northwestern.edu/publications.html. We used the Hi-C loops reported in hg38. Brain volume measurements include intracranial, hippocampal, thalamus and subiculum volume measurement. The sets of GWAS SNPs associated with these traits significantly overlap among each other (Jaccard similarity > 0.65), and therefore were merged as brain-volume-associated SNPs in this study.

We evaluated the correlations between $\Delta repression$ and SNP-SELEX scores for each TF in each tested biosample. In a biosample, TFs having at least 10 SNPs holding significant SNP-SELEX and significant $\Delta repression$ scores were included to ensure a robust estimation on the correlation between $\Delta repression$ and SNP-SELEX scores.

TF ChIP-seq data used here were downloaded from the ENCODE project (Additional file 1: Table S2). TF binding motifs were downloaded from the MEME Suite (https://meme-suite.org/meme/db/motifs). Find Individual Motif Occurrence (FIMO), with the default setting, was used to find the mappings of binding motifs in given sequences [75].

A TREDNet model is a two-phase model [19]. The pre-trained phase-one model has been deposited at 10.5281/zenodo.8161621 [76]. The phase-two models built in this study, as well as silencers and enhancers predicted by these models, are available at 10.5281/zenodo.12523205 [77].

### Supplementary Information

Additional file 1: Supplementary tables. It contains all supplementary tables.

Additional file 2: Supplementary notes and figures. It contains all supplementary notes, supplementary figures, and the legends of these figures.

Additional file 3. Peer review history.

## Data Availability

Please see the section of “ Data and tools” and Additional files.
