# Supplementary material for: The contribution of silencer variants to human diseases
Source: Genome Biol. 2024 Jul 8;25:184. doi: 10.1186/s13059-024-03328-1 (PMC11232194; doi:10.1186/s13059-024-03328-1)
Supplement: Supplementary file 2 — Additional file 2: Supplementary notes and figures. It contains all supplementary notes, supplementary figures, and the legends of these figures. [file 13059_2024_3328_MOESM2_ESM.pdf]

The contribution of silencer variants to human diseases.

Di Huang and Ivan Ovcharenko*

Intramural Research Program, National Library of Medicine, National Institutes of Health, Bethesda, MD, 20892, USA.

Table of Contents

Supplementary Notes **3**
Gene expression profiles. 3
Locus-specific enrichment of silencers and enhancers. 3
eQTLs. 3
Supplementary Figures **4**
Supplementary Fig. S1 4
Supplementary Fig. S2 5
Supplementary Fig. S3 6
Supplementary Fig. S4 7
Supplementary Fig. S5 8
Supplementary Fig. S6 9
Supplementary Fig S7 10
Supplementary Fig. S8 11
Supplementary Fig. S9 12
Supplementary Fig. S10 13
Supplementary Fig. S11 14
Supplementary Fig. S12 15
Supplementary Fig. S13 16
Supplementary Fig. S14 17
Supplementary Fig. S15 18
Supplementary Fig. S16 19
Supplementary Fig. S17 20
Supplementary Fig. S18 21
Supplementary Fig. S19 22
Supplementary Fig. S20 23
Supplementary Fig. S21 24
Supplementary Fig. S22 25
Supplementary Fig. S23 26
Supplementary Fig. S24 27

Supplementary Notes

Gene expression profiles.

We obtained the gene expression data from the ENCODE project (1) for 215 biosamples (Additional file1: Table S4). We used gene annotations from the GENCODE (2) to define the transcription start site for each gene. Candidate silencers or enhancers were associated with their nearest genes. Gene expression levels were normalized as the fold change relative to the average expressions across biosamples. For a gene (say i) and its expression level in a biosample b (say, $e_{i,b}$), its normalized expression level $ne_{i,b}$ was calculated as

$$ne_{i,b} = \log_{10} \frac{e_{i,b}}{\frac{1}{N}\sum_{k \in all\ biosamples} e_{i,k}}$$, N = the number of biosamples.

Locus-specific enrichment of silencers and enhancers.

We used gene annotations from GENCODE (2). The locus of a gene encompasses the gene body along with its two flanking upstream and downstream intergenic regions. Using this annotation, there are 26,550 distinct gene loci in the human genome. For a given gene locus (say, g) and a biosample, the count of candidate silencers located within this locus was tallied, and the silencer enrichment significance was determined using the binomial test, i.e.,

$$p(X > n) = \sum_{i=n+1}^{N} \binom{N}{i} p_g{}^{i} \left(1 - p_g\right)^{N-i}, \quad p_g = \frac{l_g}{L}, \qquad (1)$$

where n and N are the numbers of silencers within the gene locus g and in the whole genome, respectively. l_g and L denote the length of the locus g and the whole genome, respectively. Using Bonferroni multiple-testing correction, the silencer density in the locus g is regarded as significantly higher than expected in the whole genome when $p(X > n) \leq 0.05/G$. Here, G is the total number of gene loci in the whole genome. Similarly, the significance of enhancer enrichment in a gene locus is assessed based on enhancer counts.

eQTLs.

We downloaded eQTL data from the GTEx project (3) for 17 distinct tissues, comprising 13 brain tissues, colon, lung, spleen, and whole blood. For each GTEx tissue, we checked the distribution of eQTLs within candidate silencers in the corresponding biosamples. For example, we gathered eQTLs from all brain GTEx tissues and examined their density within candidate silencers in each brain biosample. In the end, 40 biosamples were tested in this analysis.

Supplementary Figures

Supplementary Fig. S1

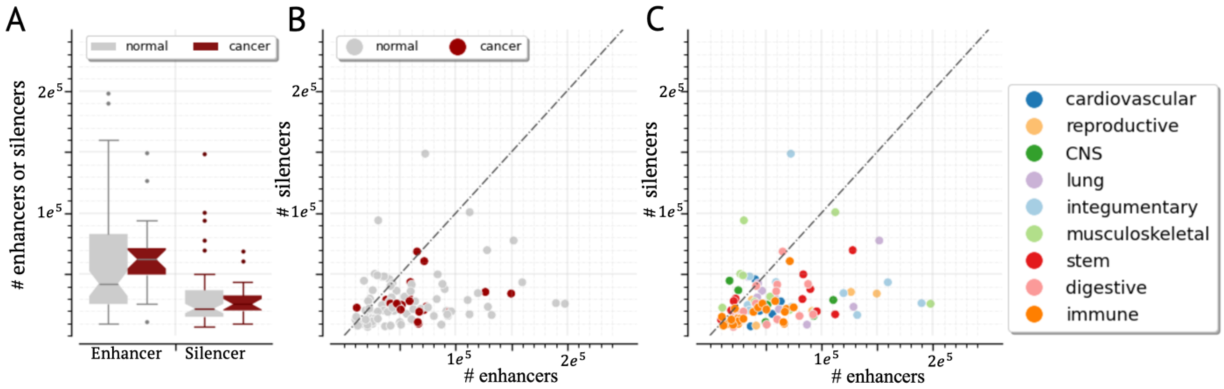

Figure S1. Numbers of candidate silencers and enhancers across biosamples. Each dot represents a biosample.

Supplementary Fig. S2

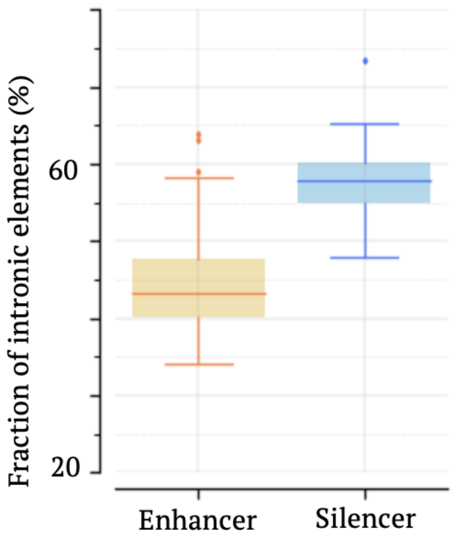

Figure S2. Fractions of intronic candidate silencers and enhancers across biosamples.

Supplementary Fig. S3

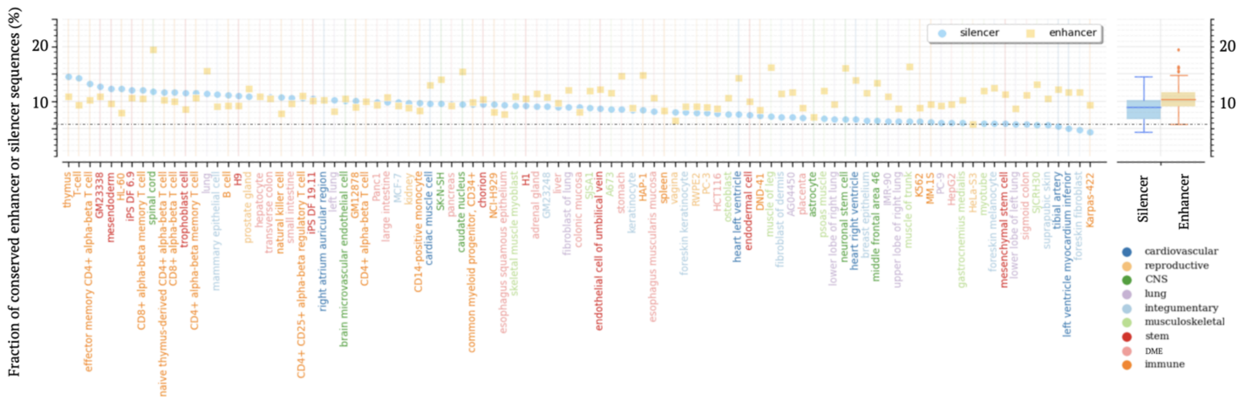

Figure S3. Fractions of conserved candidate silencers or enhancers. DME represents "digestive and metabolic and endocrine" biosample categories.

Supplementary Fig. S4

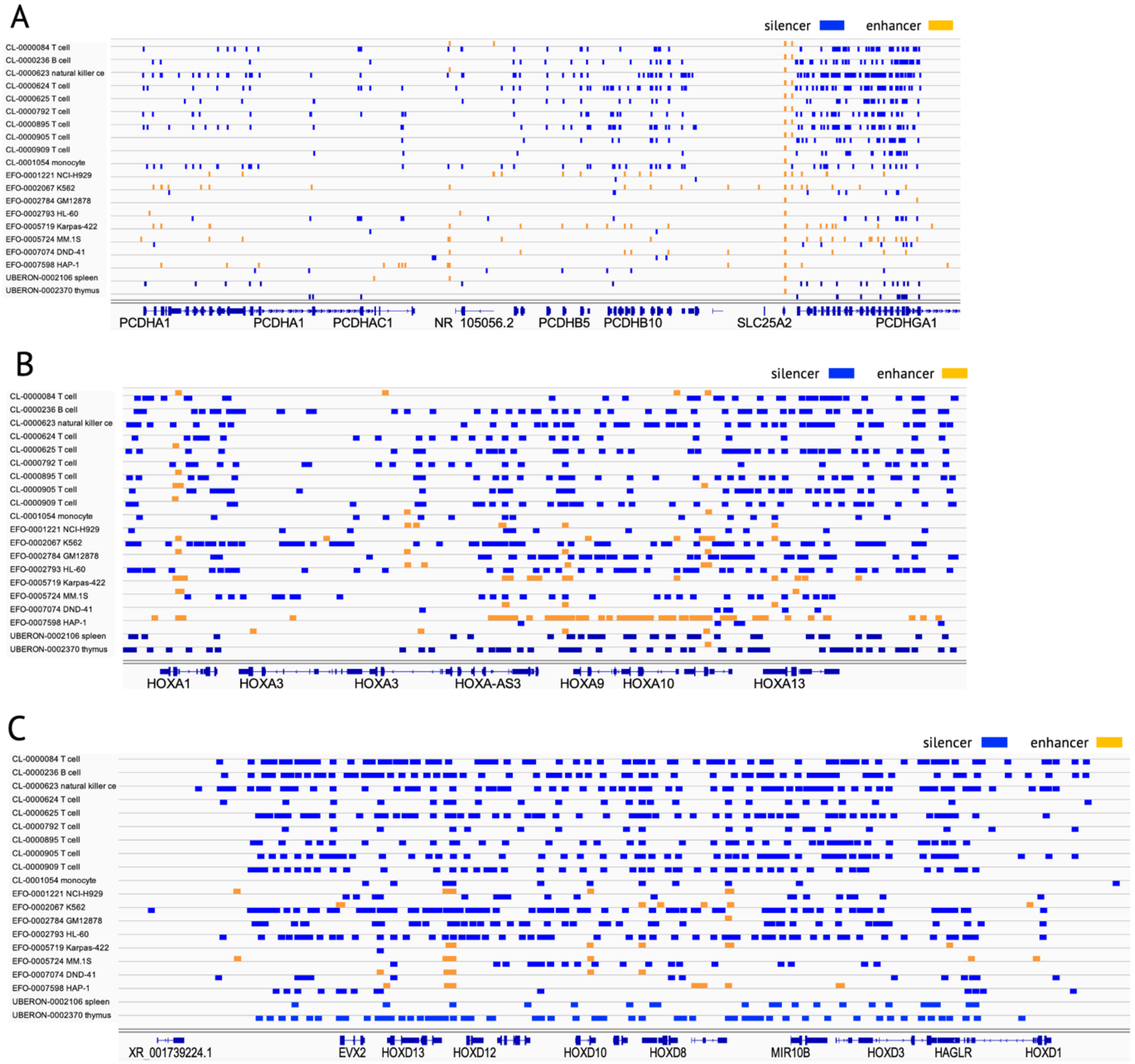

Figure S4. Profiles of candidate silencer and enhancer across immune biosamples in (A) the loci of *PCDHA/B/G* genes, (B) the loci of *HOXA* genes, (C) the loci of *HOXD* genes. All these gene loci are enriched with candidate silencers in immune biosamples.

Supplementary Fig. S5

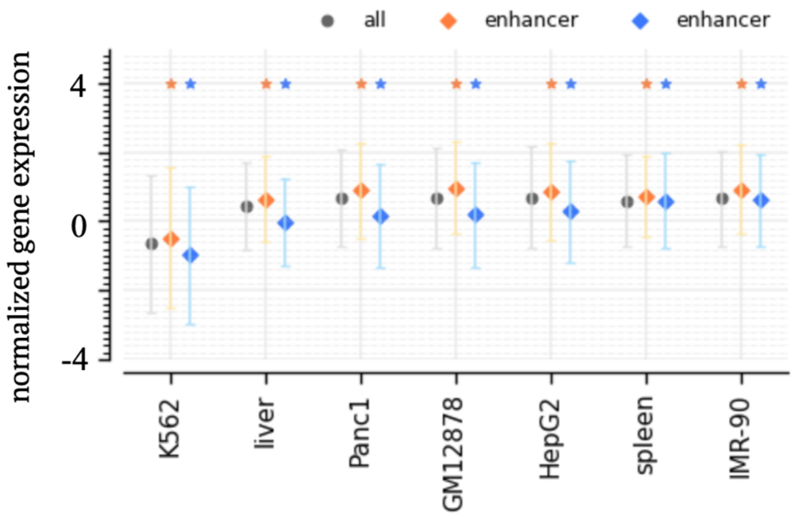

Figure S5. Expression of genes in contact with candidate silencers. The markers and their flanking lines represent the medians and standard deviations of gene expression levels. Blue and orange asterisks denote significantly low and high expression levels, respectively, compared to those of all assayed genes ($p<0.05$).

Supplementary Fig. S6

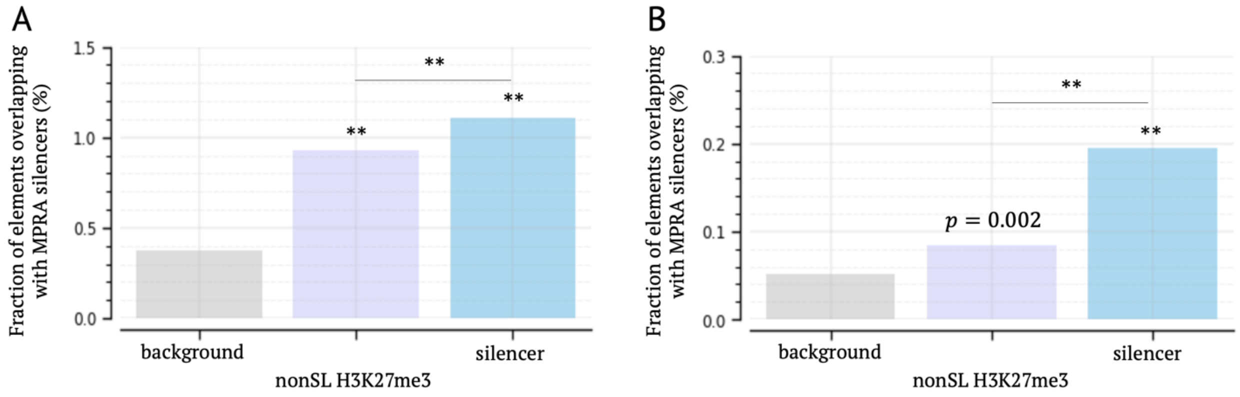

Figure S6. Significant overlap between candidate silencers and MPRA experimentally validated silencers in the biosamples (A) K562 and (B) HepG2. $**: p < 10^{-10}$. "nonSL H3K27me3" represents the H3K27me3 ChIP-seq peaks not overlapping with candidate silencers.

Supplementary Fig S7

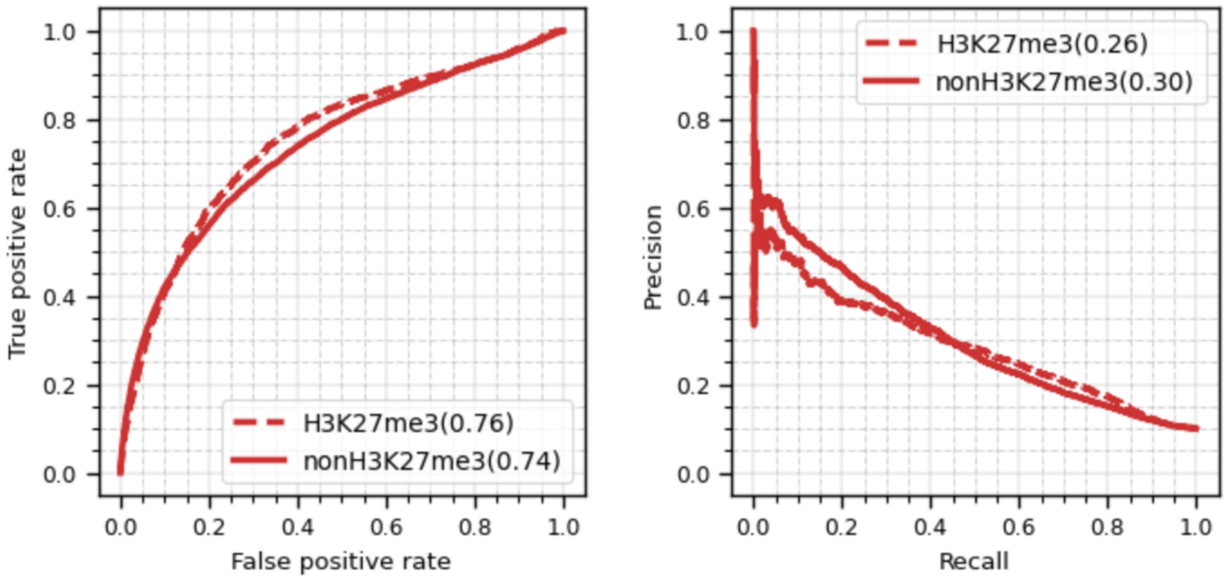

Figure S7. Classification performance of the TREDNet model on MPRA silencers with and without H3K27me3 ChIP-seq peaks.

Supplementary Fig. S8

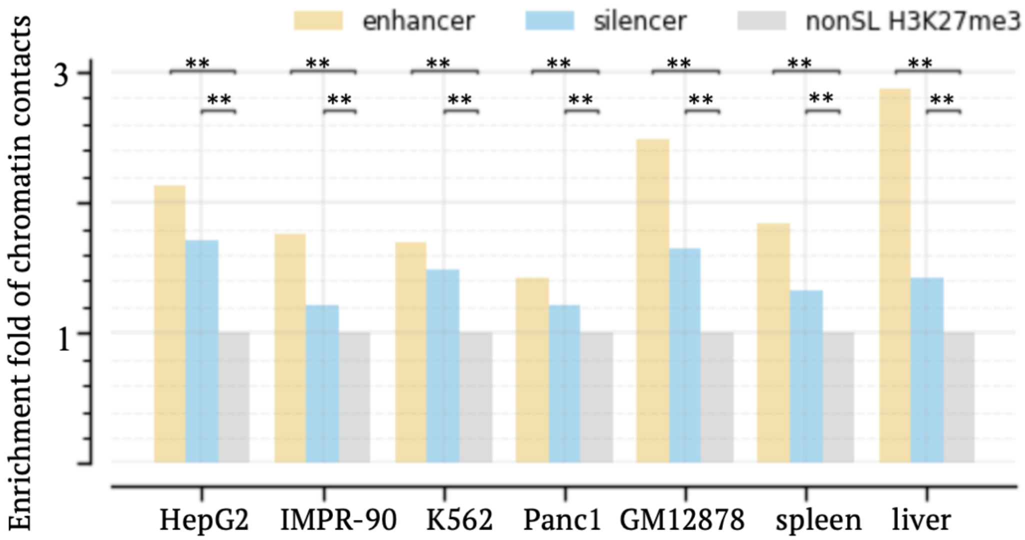

Figure S8. Enrichment of chromatin contacts in candidate silencers, enhancers and H3K27me3 ChIP-seq peaks not overlapping with candidate silencers (represented as nonSL H3K27me3). $**$: $p < 10^{-10}$.

Supplementary Fig. S9

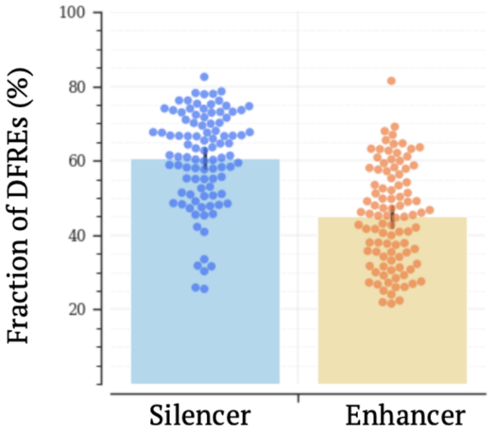

Figure S9. Fractions of DFREs among candidate silencers and enhancers across biosamples.

Supplementary Fig. S10

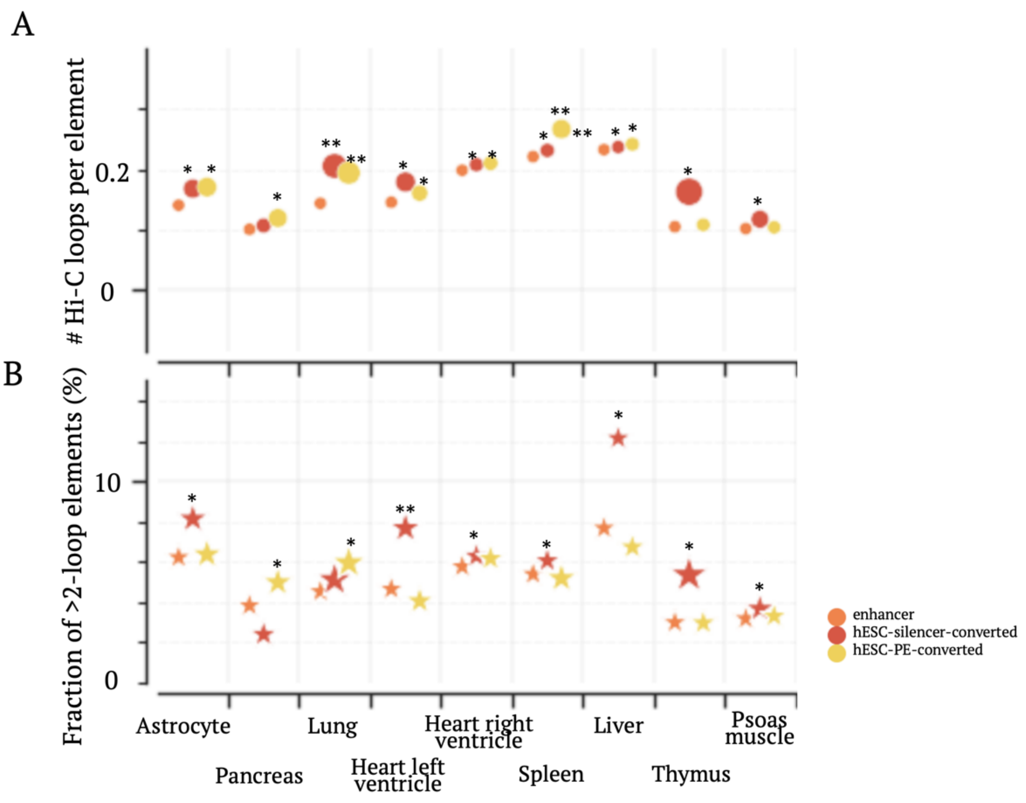

Figure S10. Density of chromatin contacts across enhancer groups. (A) Numbers of chromatin contacts per element. (B) Fractions of elements having >2 contacts. $* \, p < 10^{-3}$ and $** \, p < 10^{-8}$.

Supplementary Fig. S11

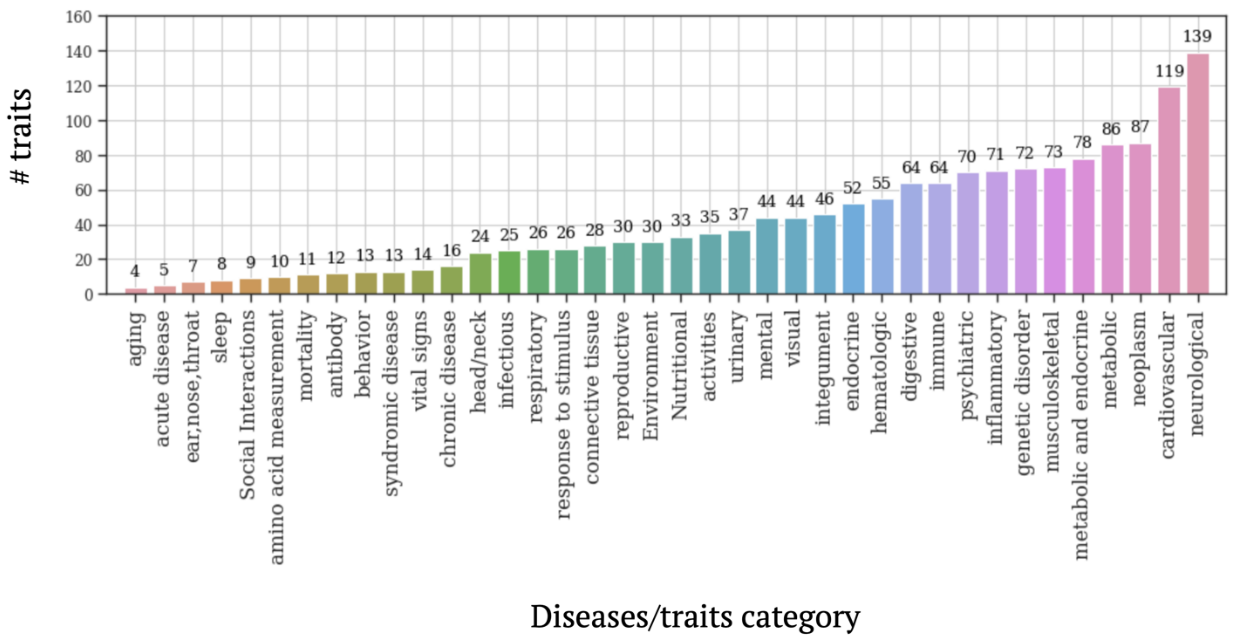

Figure S11. The distribution of GWAS traits investigated in this study.

Supplementary Fig. S12

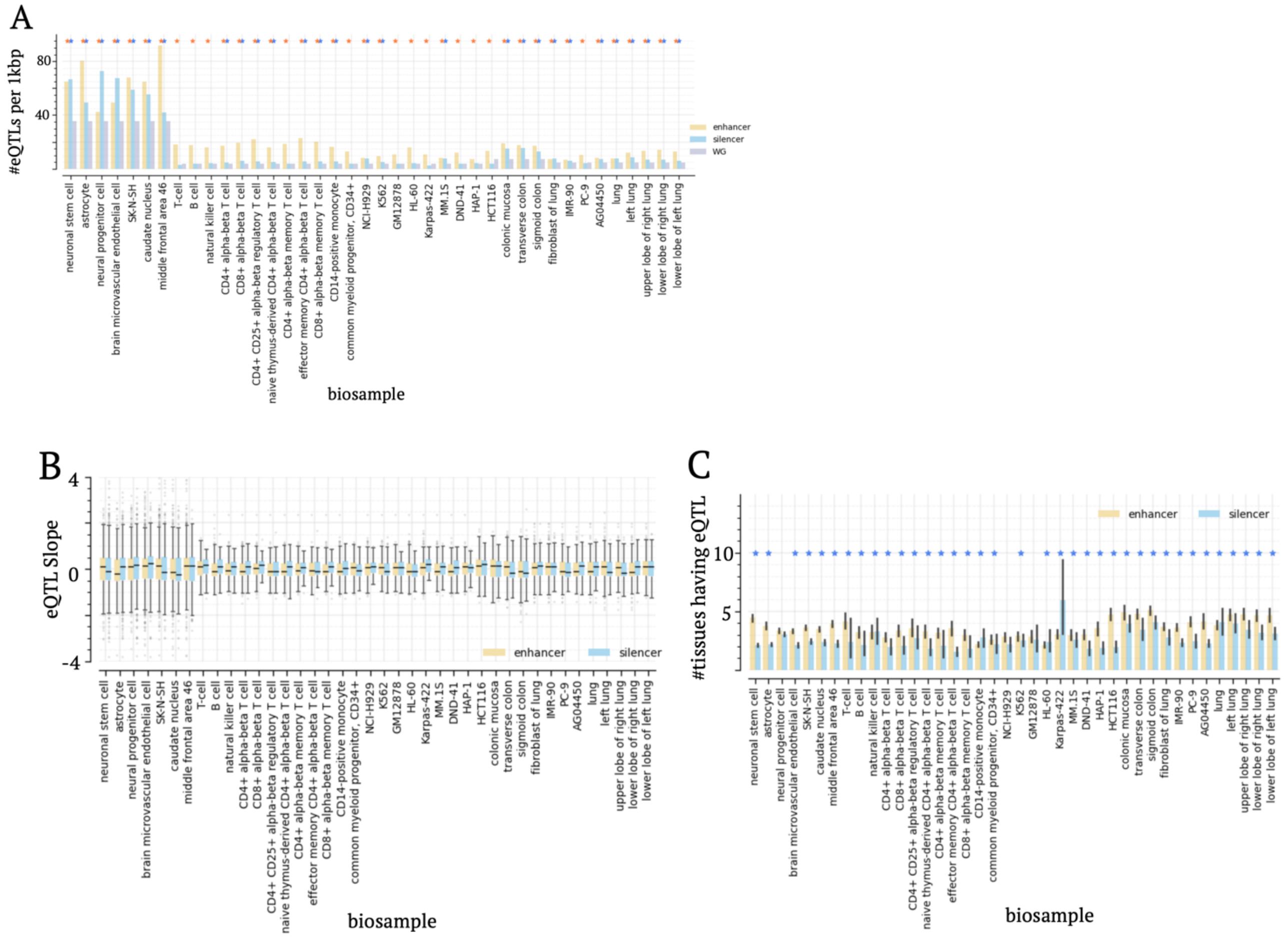

Figure S12. Distribution of eQTLs. (A) Densities, (B) eQTL slopes, and (C) tissue-specificities of eQTLs within candidate silencers and enhancers. The asterisks in (A) represent the significant enrichment compared to the whole genome ($p<0.05$). The asterisks in (C) represent the significant tissue-specificity levels of candidate silencer eQTLs compared to those of enhancer eQTLs. For a given eQTL, a low number of tissues in which this eQTL was detected suggests its high tissue specificity.

Supplementary Fig. S13

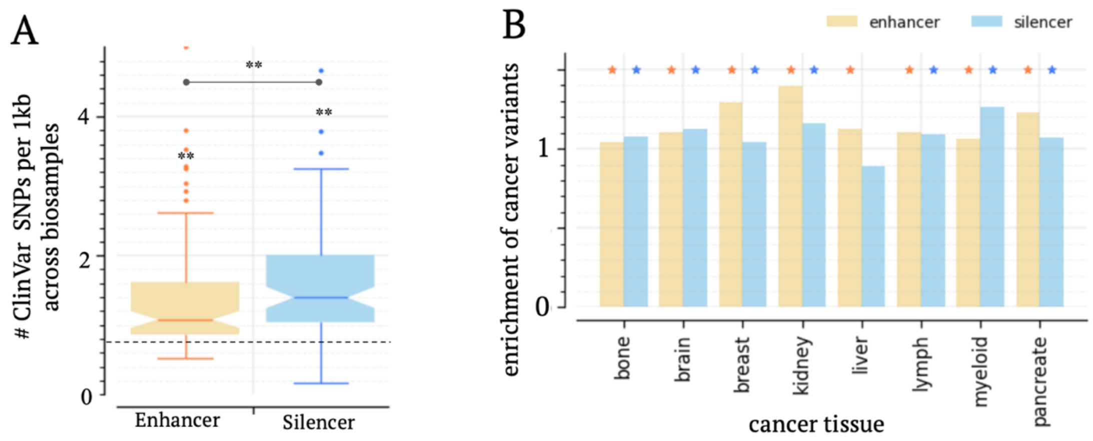

Figure S13. Enrichment of ClinVar SNPs and cancer somatic variants in candidate silencers. (A) Densities of ClinVar SNPs in candidate silencers and enhancers across all tested biosamples. The dashed line represents the density of ClinVar SNPs in the whole genome. $** \, p < 10^{-5}$. (B) Densities of cancer somatic variants within candidate silencers in matched biosamples. Blue and orange asterisks represent a significant enrichment within candidate silencers and enhancers compared to the whole genome (p<0.05), respectively.

Supplementary Fig. S14

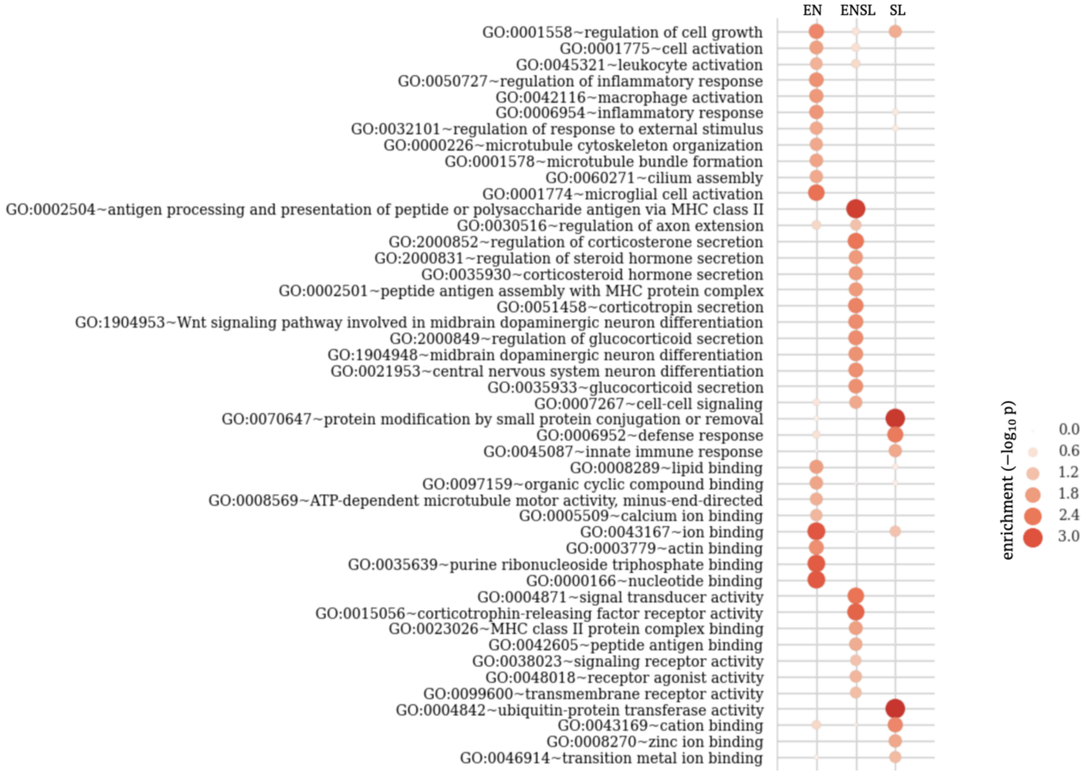

Figure S14. Function analysis of PD-associated gene groups defined in Fig. 4C.

Supplementary Fig. S15

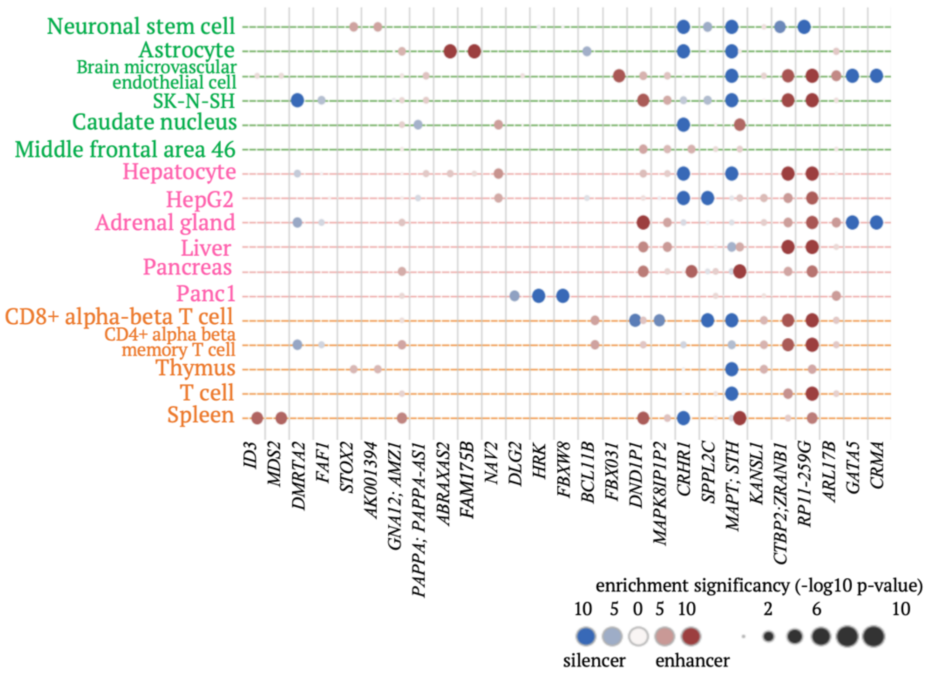

Figure S15. Enrichments of brain-volume-associated SNPs within candidate silencers and enhancers across gene loci. Gene loci having significant enrichment of the examined SNPs within either candidate silencers or enhancers are included in the plot.

Supplementary Fig. S16

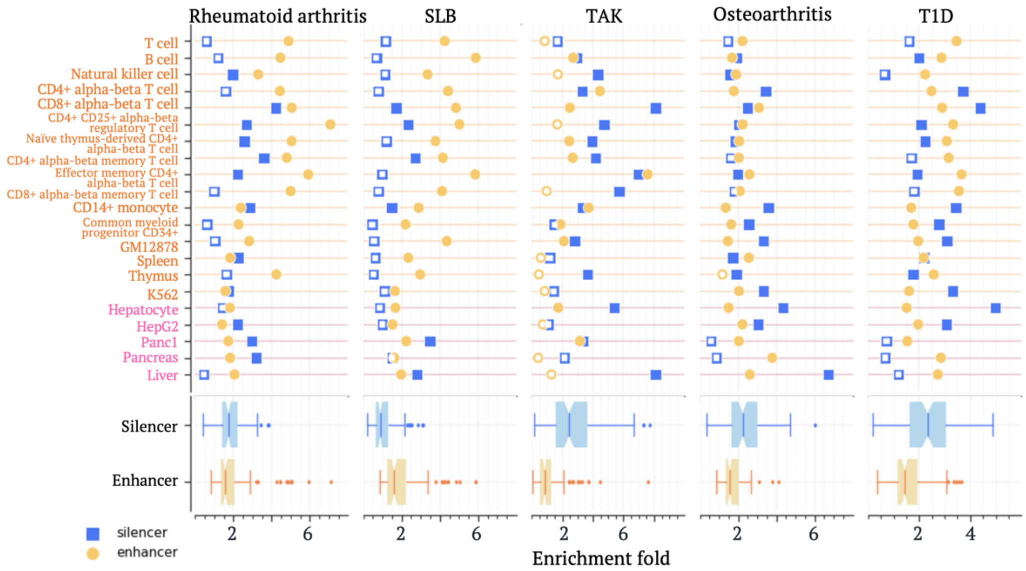

Figure S16. Enrichments of SNPs associated with autoimmune diseases. Enrichment folds are estimated in comparison to the whole genome. Significant enrichments are denoted by solid markers ($p < 10^{-5}$).

Supplementary Fig. S17

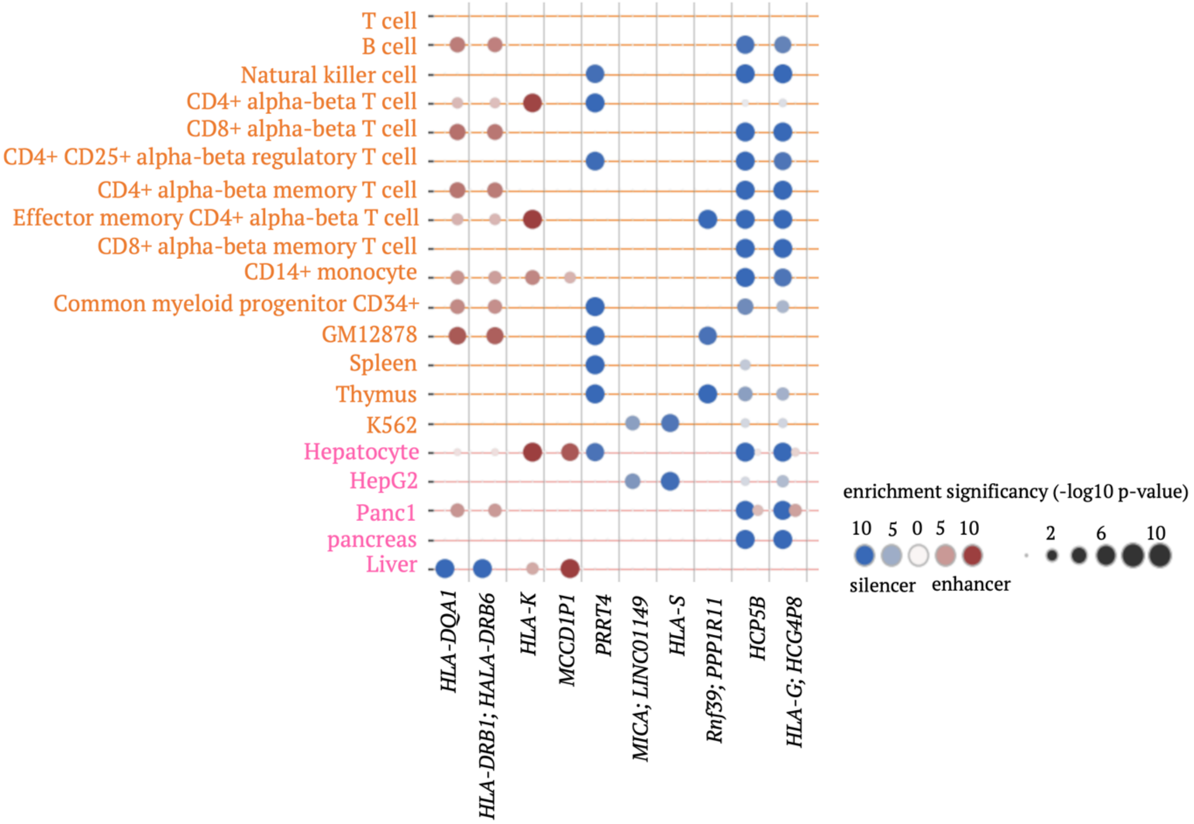

Figure S17. Enrichments of TAK-associated SNPs within candidate silencers and enhancers in gene loci. Gene loci having significant enrichment of the examined SNPs within either silencers or enhancers are included in the plot.

Supplementary Fig. S18

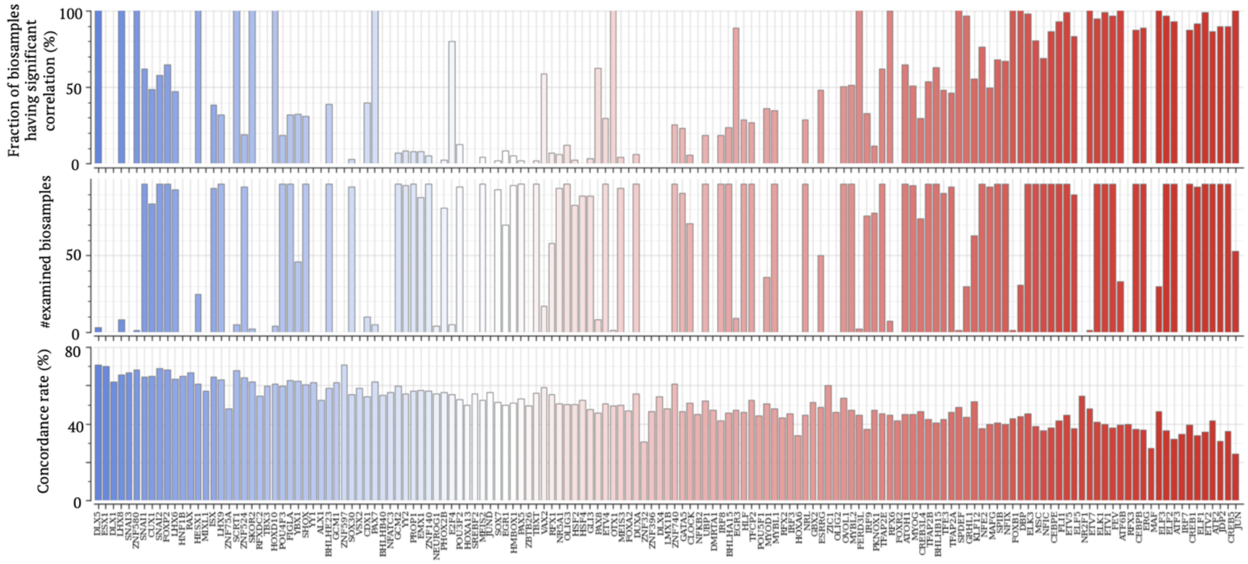

Figure S18. Distribution of significant correlations between $\Delta repression$ and SNP-SELEX scores for each TF across biosamples. For each TF, the top panel presents the number of biosamples for which SNP-SELEX scores of this TF significantly correlate with $\Delta repression$. The middle panel presents the number of biosamples for which this TF was examined. The bottom panel presents the concordance rate between $\Delta repression$ and SNP-SELEX scores.

Supplementary Fig. S19

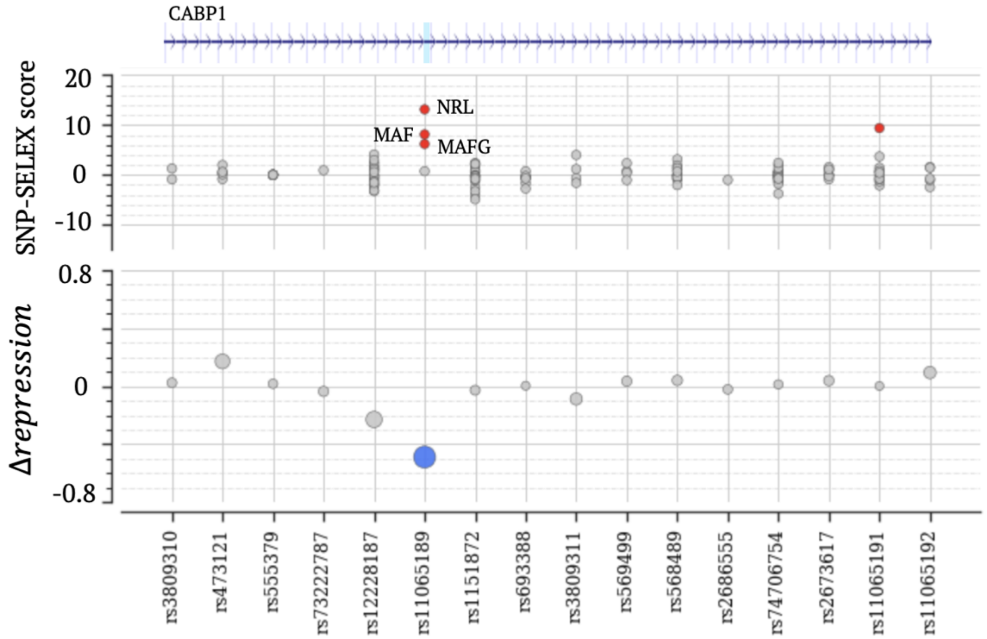

Figure S19. $\Delta repression$ and SNP-SELEX scores on the SCZ-associated rs11065189 and its neighboring SNPs. In the top panel, red/grey dots indicate significant/insignificant SNP-SELEX scores. TFs corresponding to the significant scores are listed. In the bottom panel, blue/grey dots indicate significant/insignificant $\Delta repression$ scores.

Supplementary Fig. S20

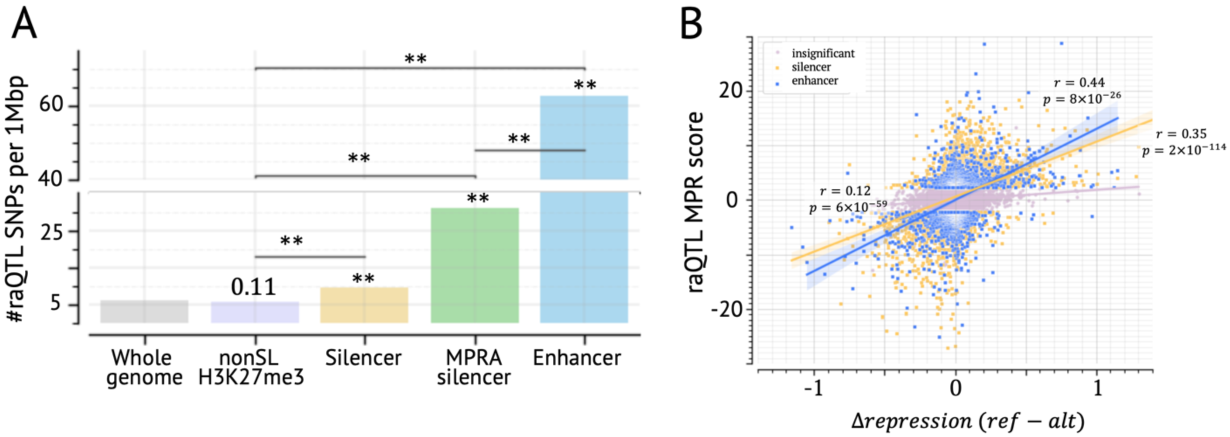

Figure S20. Analyses based on raQTLs. (A) raQTLs are enriched in the predicted silencers and MPRA silencers as compared to the expected across the whole genome and within non-predicted-silencer H3K27me3 ChIP-seq peaks (labeled as nonSL H3K27me3 here). Asterisks and the number over the bars suggest the enrichment p value as compared to the whole genome. $**: p < 10^{-10}$. (B) $\Delta repression$ scores significantly correlate with raQTL scores, regardless of candidate silencers or enhancers.

Supplementary Fig. S21

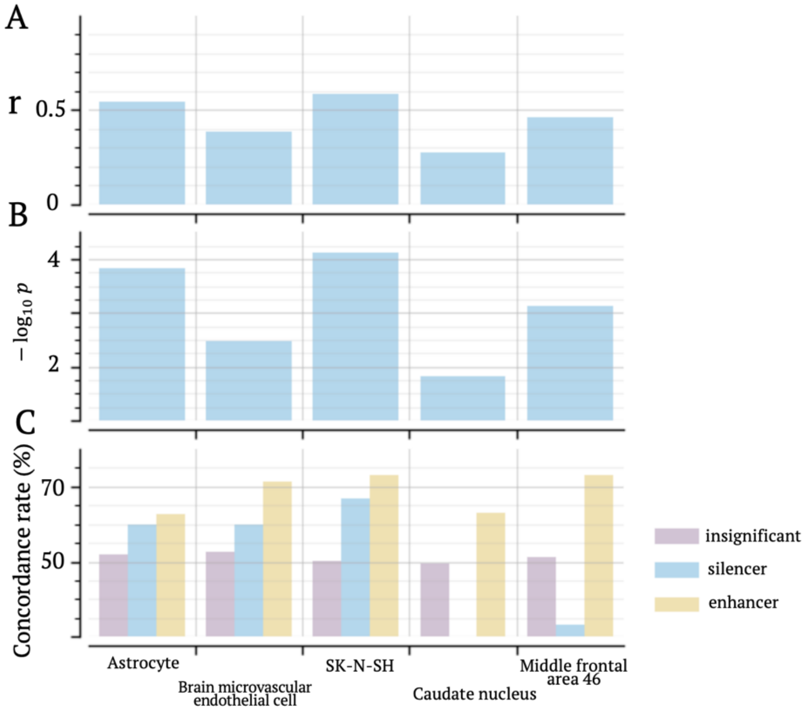

Figure S21. Correlations between $\Delta repression$ and dementia MPRA scores across brain biosamples. (A) correlation coefficients between $\Delta repression$ and dementia MPRA scores. (B) significant p values these coefficients. (C) concordance rates between $\Delta repression$ and dementia MPRA scores in three SNP categories: insignificant-$\Delta repression$ SNPs, significant-$\Delta repression$ silencer/enhancer SNPs.

Supplementary Fig. S22

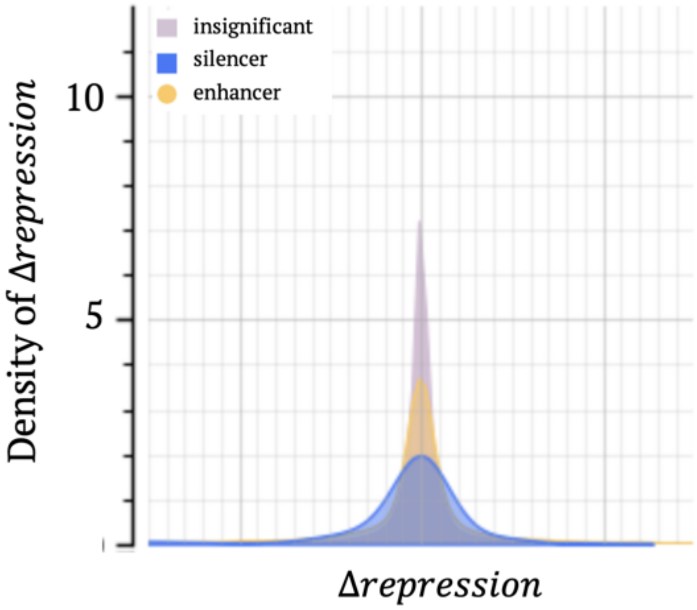

Figure S22. $\Delta repression$ score distributions for different SNP groups. SNP groups here are those having insignificant mMPRA scores, significant mMPRA scores in candidate silencers, and in candidate enhancers.

Supplementary Fig. S23

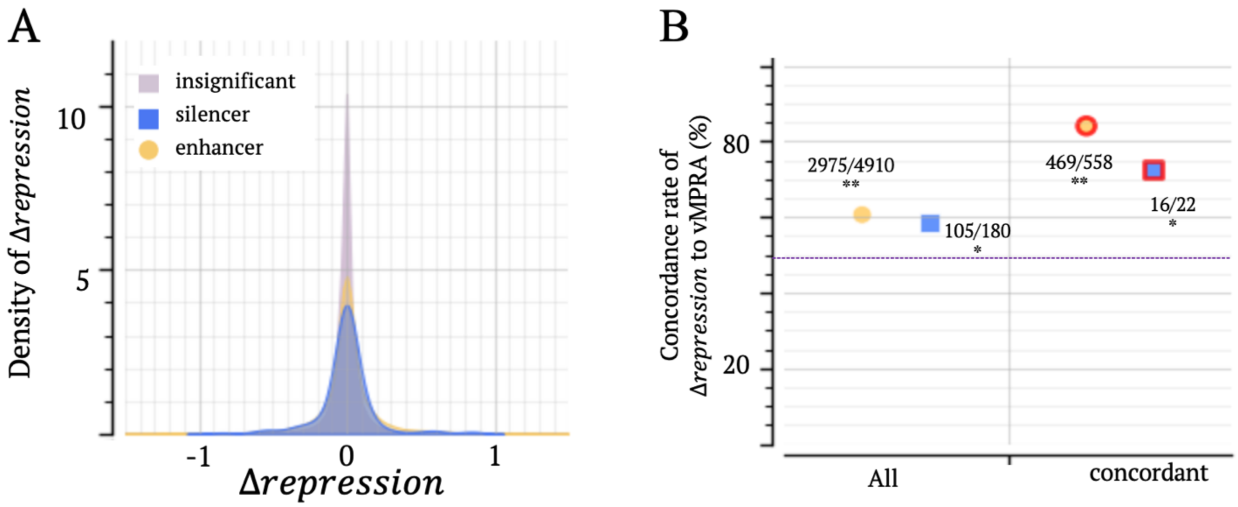

Figure S23. Correlation between $\Delta repression$ and vMPRA scores. (A) Distribution of $\Delta repression$ score across SNP groups. SNP groups here are those having insignificant vMPRA scores, and significant vMPRA scores in candidate silencers and enhancers. (B) Directional concordance between $\Delta repression$ and vMPRA scores. "All" represents all significant-vPRA SNPs in candidate silencers or enhancers, while "concordant" represents the SNPs where significant mMPRA and vMPRA scores directionally align. The dashed line represents the expectation after randomly shuffling $\Delta repression$. $** p < 10^{-8}$, $* p < 0.01$

Supplementary Fig. S24

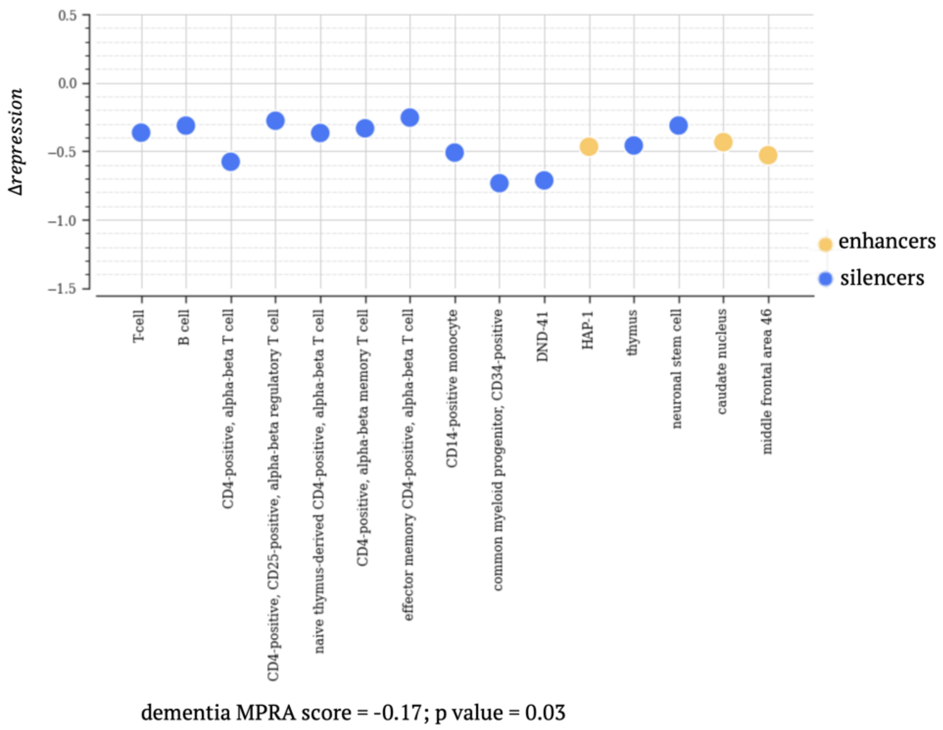

Figure S24. $\Delta repression$ scores at rs242561. The biosamples where rs242561 is located within a candidate silencer or enhancer are included.

1. Luo Y, Hitz BC, Gabdank I, Hilton JA, Kagda MS, Lam B, et al. New developments on the Encyclopedia of DNA Elements (ENCODE) data portal. Nucleic Acids Res. 2020;48(D1):D882-d9.
2. Frankish A, Diekhans M, Jungreis I, Lagarde J, Loveland Jane E, Mudge JM, et al. GENCODE 2021. Nucleic Acids Research. 2021;49(D1):D916-D23.
3. The GTEx Consortium. The Genotype-Tissue Expression (GTEx) pilot analysis: Multitissue gene regulation in humans. Science. 2015;348(6235):648-60.
