# Supplementary material for: The contribution of silencer variants to human diseases
Source: Genome Biol. 2024 Jul 8;25:184. doi: 10.1186/s13059-024-03328-1 (PMC11232194; doi:10.1186/s13059-024-03328-1)
Supplement: Supplementary file 3 — Additional file 3. Peer review history. [file 13059_2024_3328_MOESM3_ESM.docx]

**Review history**

**First round of review**

**Reviewer 1**

In this work, Huang and Ovcharenko attempt to study the contribution of silencer mutations to human diseases. They first identify putative silencers and enhancers by applying a deep learning algorithm (TREDNet) to ChIP-seq data across many human cell types. Subsequently, they used these datasets to compare the functional relevance of identified silencers and enhancers. Finally, they explored the link between silencers and diseases using available GWASs. They showed that silencers were associated with distinct diseases and have significative overlap with GWAS-SNPs affecting either transcription factor binding or cis-regulatory activity, as assessed by published SNP-SELEX and MPRA experiments, respectively.

This work represents a comprehensive study of silencers in the context of disease providing potential insights towards a better characterization of regulatory variants. However, a critical concern of the study comes from the lack of functional characterization of predicted silencers, which is solely based on H3K27me3 enrichment. Moreover, the contribution of genetic variation associated with silencers requires further experimental validation in order to support their claims.

Main remarks
1) In this study, silencer identification primarily relies on H3K27me3 ChIP-seq peaks. While this histone mark is a well-characterized and widely-accepted proxy of repressive regulatory
elements (i.e., silencers), not all H3K27me3 peaks might represent active silencers. Likely, a substantial (if not the majority) of H3K27me3 enriched regions might simply represent repressive chromatin states of otherwise "activatory" regulatory elements, such as enhancers and promoters. Therefore, without assessment of silencer activity by, for instances, high-throughput reporter assays, the identified regions should not be called silencers but rather repressed chromatin regions.

2) The authors used the deep learning model TREDNet to identify putative silencers and enhancers. However, this model was not originally designed to identify silencers. How the accuracy of the model was assessed is not clearly described in the Methods. If the authors used H3K27me3 peaks as a proxy for silencers, it might represent a circular analysis. In any case, the prediction model should be validated by experimental data confirming the actual silencer activity of predicted "silencers".

3) Based on the above two points, the authors might consider whether renaming the identified "silencers" as "repressed chromatin" might be more accurate.

4) The authors used published MPRA data to assess the regulatory impact of silencer-overlapping SNPs. However, these MPRAs have not been designed to assess silencer activity. Therefore, the relevance of these findings is mitigated by the fact that silencer activity is not properly assessed in these assays. They should perform dedicated experiments to functionally assess the impact of genetic mutations on the silencer activity itself.

5) To support the functional relevance of the study, the authors should provide experimental assessment of disease-associated silencers by performing CRISPR-mediated deletion or mutation of silencer candidates in relevant cell lines.

6) In addition to focusing on specific diseases, the full list of GWAS traits significantly enriched with silencers or enhancers should be provided.

7) Figure 7C-D: the lead as well as the relevant LD-SNPs should be highlighted in panel C

8) Introduction (Lines 53-56). When mentioning the recent advancements in massively parallel reporter assays (MPRAs) to map silencers genome-wide, additional references should be included: Doni Jayavelu et al. 2020, Hussain et al. 2023 and Xiusheng et al. 2023.

9) In general, the figures contain too much information and make sometime difficult to extract the relevant information. Simplification, moving some panels to supplemental figures or more specifically chosen examples to support the most important conclusions could be beneficial to the legibility.

10) Line 71: "accuracy of silencer prediction was on par with our prior model". More information on the difference between the present and prior model could be beneficial to understanding the significance of the 0.84 AUC ROC, and why we should prefer a second model when it gives no improvement in AUC ROC compared to the prior model.

11) Line 104-107: For the 3 loci mentioned, PCDH, HOXA and HOXD, with enrichment in silencers, It would important to see a screenshot of these loci showing the predicted enhancer and silencers.

12) Line 133/Figure 2E: figure legend is missing in the text. What does the dotted line mean, and how the number of 71.4% of CNS cells that are mentioned in the text (Line 133) to have dense silencer populations can be deduced? Is this percentage to be found in the figure?

Typos
line 43: isolators = insulators
Line 70: "building a multi-label classifier for each of biosamples collected …" should be "building a multi-label classifier for each of the biosamples collected …" .
Line 105: "Display" should be "Displays"
Line 112: "although not comprehensively enough" should be "although not comprehensive enough".
Line 133: "display" should be "displays"
Line 191: "2.2 million GWAS SNPs were complied" should be "2.2 million GWAS SNPs were compiled"
Line 227: "underlaying" should be "underlying"
line 245: nature killer = natural killer
line 294: "pervious"= should be "previous"
line 360: provide year of publication for reference "Cooper et al."
line 462: "the disruption GABAergic interneuron » should be "the disruption of the GABAergic interneuronS"
line 452: "majority" should be "the majority"
line 452: "are DFREs" should be "as DFREs"
line 453: "which still can be elevated" should be "which could still increase"
line 216: The "locus TLR9" is not displayed in Figure 4C.
Figure 4D: Asterixis missing to denote significance mentioned in line 242?
Figure 6D: TF gene names are illegible.

**Reviewer 2**

Huang et al. presented an interesting study that explored the potential functions of genetic variants in non-coding silencer elements. By first making a deep learning prediction on silencers and enhancers, they further studied the enrichment of GWAS SNPs in these two non-coding elements. They identified a few examples indicating the causal effects of SNPs within silencers in different diseases. The study is sound, and the knowledge gained is important, as silencers have not been studied broadly, and most of the time, only enhancers were considered and studied when interpreting genetic variants in the human population.

There are some points that may improve the manuscript.
1. How many predicted silencers overlap with the experimentally identified silencers?
2. It would be interesting to integrate the eQTL data to check if the genetic changes in silencers would affect the nearby genes.
3. Have the authors also compared their analysis with the ClinVar data to see any more diseases related variants fall within silencers? Or other cancer related database with disease-associated mutations.
4. The authors seem to use SNP and mutations interchangeably. Would be better to clarify the definition of these two events.
5. The manuscript will benefit from better editing of the figures and more detailed figure legends.

Minor
Line 43: isolators - insulators
Fig. 1C. It is complicated to have 3 subgraphs in 1 panel, which is hard to understand the data.
Fig. 2A. There are no units on the y-axis.
Fig. 7. A few motifs related to SNPs have no explanation in the legend.

**Authors’ response to reviewers**

Reviewer #1: Review Genome Biology Silencers

In this work, Huang and Ovcharenko attempt to study the contribution of silencer mutations to human diseases. They first identify putative silencers and enhancers by applying a deep learning algorithm (TREDNet) to ChIP-seq data across many human cell types. Subsequently, they used these datasets to compare the functional relevance of identified silencers and enhancers. Finally, they explored the link between silencers and diseases using available GWASs. They showed that silencers were associated with distinct diseases and have significative overlap with GWAS-SNPs affecting either transcription factor binding or cis-regulatory activity, as assessed by published SNP-SELEX and MPRA experiments, respectively. This work represents a comprehensive study of silencers in the context of disease providing potential insights towards a better characterization of regulatory variants. However, a critical concern of the study comes from the lack of functional characterization of predicted silencers, which is solely based on H3K27me3 enrichment.

Moreover, the contribution of genetic variation associated with silencers requires further experimental validation in order to support their claims.

We would like to thank this reviewer for the positive feedback and the improvement suggestions, which were invaluable in preparing the revised version of the manuscript, including sourcing several additional experimental validation datasets to confirm the active silencing nature of the TREDNet silencers. In particular, as detailed below in our point-by-point response, we have evaluated predicted silencers and TREDnet models using MPRA validated silencers and the results from MPRA platforms designed to evaluate the silencing and activating impact of genomic regions.

Main remarks

1) In this study, silencer identification primarily relies on H3K27me3 ChIP-seq peaks. While this histone mark is a well-characterized and widely-accepted proxy of repressive regulatory elements (i.e., silencers), not all H3K27me3 peaks might represent active silencers. Likely, a substantial (if not the majority) of H3K27me3 enriched regions might simply represent repressive chromatin states of otherwise "activatory" regulatory elements, such as enhancers and promoters. Therefore, without assessment of silencer activity by, for instances, high- throughput reporter assays, the identified regions should not be called silencers but rather repressed chromatin regions.

This is a very important point, and we would like to thank the reviewer for raising this concern. To address active repression by the predicted silencers (as opposed to a heterochromatic state of the enclosing chromatin, for example), we conducted an activity analysis of predicted silencers from three perspectives.

First, we utilized the results of MPRA experiments across three biosamples: K562, HepG2, and GM12878 suitable for active silencing activity analysis. These particular MPRA experiments were specifically designed to measure both the silencing and activating effects of genomic regions. In K562 and HepG2 cells, predicted silencers frequently display negative Sharpr-MPRA scores (Ernst et al.

2016). These scores are significantly lower than those observed in enhancers and all regions tested (Figure R1). Similarly, in GM12878, there is a notable significant enrichment of negative ATAC- STARR-seq scores (Hansen and Hodges 2022) among predicted silencers compared to all tested DNA sequences and enhancers, supporting their silencing function exerted on the target gene (Figure R1).

Figure R1 MPRA scores of predicted silencers and enhancers in three biosamples. **, 𝑝 < 10^!"#^.

Additionally, to examine the regulatory impact of predicted silencers, we explored gene expression across 66 biosamples, for which gene expression profiles are available from the ENCODE project. In each examined biosample, we observed a significantly lower expression of genes proximal to predicted silencers (p<0.05, Figure R2A). Furthermore, genes in active contact with predicted silencers, as determined by Hi-C chromatin loops, exhibit significantly lower expression compared to other genes (Figure R2B).

Figure R2 illustrates the expression of genes (A) near and (B) in contact with predicted silencers. The markers and their flanking lines represent the medians and standard deviations of gene expression levels. Blue and orange asterisks denote significantly low and high expression levels, respectively, compared to all assayed genes (p<0.05).

Finally, to determine whether predicted silencers function as active silencing elements or as regions of repressed chromatin, we analyzed the abundance of transcription factor (TF) binding events and chromatin contacts, with the assumption that repressed chromatin regions would have significantly fewer TF ChIP-seq peaks and chromatin contacts. Across the biosamples with ChIP-seq data for more than 50 TFs documented by the ENCODE project, TF ChIP-seq peak densities within predicted silencers were, on average, 3.5 times those within non-predicted silencer H3K27me3 ChIP-seq peaks

(Figure R3A). Additionally, Hi-C chromatin contact densities of predicted silencers were 1.5 times those of non-predicted-silencer H3K27me3 peaks (Figure R3B). These consistent results suggest a pronounced active state of predicted silencers compared to H3K27me3 ChIP-seq peaks not predicted as silencers, supporting the effectiveness of our models in identifying active silencers.

Figure R3 Evaluation of activeness of predicted silencers. (A) TF ChIP-seq peak density and (B) enrichment of chromatin contacts in predicted silencers, enhancers and H3K27me3 ChIP-seq peaks not overlapping with predicted silencers (represented as nonSL H3K27me3). ∗: 𝑝 < 0.05 and ∗∗: 𝑝 < 10^!"#^.

We have added these results as follows (Pages 6-8).

*“Functional evaluation of silencer predictions.*

*To assess the impact of candidate silencers, we initially analyzed the expression of genes located near these elements across 66 biosamples with available gene expression profiles from the ENCODE project (see Supplementary Notes) since genes associated with active silencers are likely to be lowly expression. Across all examined biosamples, genes neighboring candidate silencers exhibit significantly lower expression than all assayed genes (*𝑝 < 0.05*, Figure 1D). Similarly, genes targeted by candidate silencers, as determined by Hi-C chromatin loops (Salameh et al. 2020), consistently display notably low expression across all tested biosamples (*𝑝 < 0.05*, Figure S5).*

*Furthermore, we directly evaluated the activity of candidate silencers by utilizing the experimental results from MPRA platforms designed to measure silencing or activating impact of genomic regions. In K562 and HepG2 biosamples, candidate silencers frequently exhibit negative scores reported by the Sharpr-MPRAs (Ernst et al. 2016). These scores are significantly lower than those observed in enhancers and all tested regions (Wilcoxon rank-sum test* 𝑝 ≤ 0.05*, Figure 1E), supporting the active silencing function of candidate silencers. Similarly, in GM12878, significant negative ATAC-STARR-seq scores, which represent “silent” genomic sequences (Hansen and Hodges 2022), are enriched among candidate silencers (*𝑝 = 4 × 10^!"#^ *vs all tested sequences, Figure 1E).*

*……*

*To further investigate whether candidate silencers actively suppress gene expression as opposed to being genomic regions of repressed chromatin, we analyzed the abundance of transcription factor binding sites (TFBSs) and chromatin contacts, under the assumption that repressed chromatin regions host significantly fewer TFBSs and chromatin contacts than active enhancer and silencer regions. In each of tested biosample with ChIP-seq data for more than 50 TFs available from the ENCODE project, candidate silencers contain, on average, 3.5 times as many TF ChIP-seq peaks as H3K27me3 ChIP-seq peaks lacking candidate silencers (Wilcoxon rank-sum test* 𝑝 < 10^!"$^*, Figure 1E).*

*Additionally, the density of Hi-C chromatin contacts within predicted silences is 1.5 times greater than the corresponding density within H3K27me3 ChIP-seq peaks lacking candidate silencers (binomial test* 𝑝 < 0.05*, Figure S8).*

*Overall, these results support that the TREDnet predicted silencers predominantly act as active silencers and not simply heterochromatic regions of the genome.”*

2) The authors used the deep learning model TREDNet to identify putative silencers and enhancers. However, this model was not originally designed to identify silencers. How the accuracy of the model was assessed is not clearly described in the Methods. If the authors used H3K27me3 peaks as a proxy for silencers, it might represent a circular analysis. In any case, the prediction model should be validated by experimental data confirming the actual silencer activity of predicted "silencers".

We apologize for the insufficient description of the method (corrected in the revised version of the manuscript) and completely agree with the reviewer that the validation of TREDNet silencers has to be performed using independent experimental data. While the validation in the original manuscript was indeed based on such data, we significantly expanded the testbed of TREDNet silencer validations by collecting a large number of additional independent experimental validation datasets. In particular, we have further evaluated the performance of TREDnet models on silencers experimentally detected in MPRA studies. We compiled a total of 7,701 MPRA silencers from two studies: ReSE (Pang and Snyder 2020) and STARR-seq (Doni Jayavelu et al. 2020). These MPRA silencers, detected not based on H3K27me3 signals, can provide an independent evaluation on TREDnet models.

Within these MPRA silencers, 6,999 have no overlap with the sequences used to train the TREDnet in K562. On this subset of 6,999 MPRA silencers, the TREDnet achieved a classification performance of AUROC = 0.74 and AUPRC = 0.30 with a 1:9 ratio of positive to control samples. It notably outperforms our prior SVM model (Huang et al. 2019)(AUROC = 0.63 and AUPRC = 0.18, Figure R4) and the general H3K27me3 signal profile (AUROC = 0.58 and AUPRC = 0.16, Figure R4). It shows marginal improvement over our prior CNN model (AUROC = 0.75 and AUPRC = 0.24). These findings indicate the TREDnet’s effectiveness in detecting silencers.

Figure R4 Classification performance on MPRA silencers. (A) comparison of the TREDnet with the CNN and H3K27me3 ChIP-seq peaks. (B) performance of the TREDnet on MPRA silencers with and without H3K27me3 signals.

We summarized the results of these analyses in the manuscript as follows (Page 7).

*“……, we validated the TREDnet silencer model on an independent experimental dataset of MPRA silencers. After excluding MPRA silencers overlapping sequences used for training the TREDnet model, we had 6,999 K562 MPRA silencers remaining for validation. On this subset of MPRA silencers, the TREDnet model demonstrates a classification performance of AUROC = 0.74 and AUPRC = 0.30 with the 1:9 ratio of positive to control samples. It shows a marginal improvement over our prior CNN classifier (Huang and Ovcharenko 2022) and significantly outperforms our prior SVM model (Huang et al. 2019), and general H3K27me3 signal profiles (Figure 1G). Furthermore, the TREDnet silencer model can effectively distinguish both H3K27me3 and non-H3K27me3 MPRA silencers from control sequences (Figure S7). These results reaffirm that the TREDnet silencer model can identify active silencers with respectable accuracy.”*

In addition to the independent experimental validation, the TREDNet silencer (and enhancer) model was properly trained using cross-validation and a “hold out” dataset for testing (Hudaiberdiev et al. 2023). Specifically, human chromosomes 7 and 8 were never used in the model training and were exclusively used for testing (Hudaiberdiev et al. 2023). The results on these testing sequences are presented in Figure 1A. Consequently, in addition to the independent experimental validation, the training of TRENDnet models was not circular and was performed with proper hold-out testing datasets.

For clarification, we added the following clarification to the Methods section (Page 29):

*“We held out chromosomes 7 and 8 for testing. All other autosomes were used for building the classification model (Hudaiberdiev et al. 2023). Consequently, testing sequences, having no overlap with training sequences, provide an unbiased computational evaluation on the performance of the TREDnet models.”*

3) Based on the above two points, the authors might consider whether renaming the identified "silencers" as "repressed chromatin" might be more accurate.

We understand the concerns raised by the reviewer. The newly included validation results (Figures R1- 4) demonstrate that predicted silencers frequently function as active “silencing” elements, rather than repressed chromatin regions, and that TREDnet models can accurately distinguish experimentally validated MPRA silencers from controls. Nevertheless, since the experimental validation is available for a subset of TREDNet silencers (albeit across several biosamples), we refer to predicted silencers as “candidate silencers” in the revised manuscript to underscore the limitation of our study and to avoid overexaggerating presented results.

For example (Page 8):

*“… …, these results support that the TREDnet predicted silencers predominantly act as active silencers and not simply heterochromatic regions of the genome. Therefore, we refer to them as candidate silencers.”*

4) The authors used published MPRA data to assess the regulatory impact of silencer-

overlapping SNPs. However, these MPRAs have not been designed to assess silencer activity. Therefore, the relevance of these findings is mitigated by the fact that silencer activity is not properly assessed in these assays. They should perform dedicated experiments to functionally assess the impact of genetic mutations on the silencer activity itself.

We acknowledge the concerns raised by the reviewer regarding the suitability of the MPRA experiments used here for assessing silencer variants. However, we would like to note that several recent MPRA studies have been specifically designed to detect silencer (and/or enhancer) activity by avoiding the use of minimal promoters, in particular. In addition to the detailed MPRA analysis presented in response to the earlier queries of the reviewer, we also discovered a notable number of MPRA SNPs situated within predicted silencers as well as MPRA validated silencers. For example, in K562 cells, raQTLs were found to exert a significant difference in regulatory influence between alleles (van Arensbergen et al. 2019). These raQTL variants are enriched in MPRA validated silencers as well as predicted silencers compared to the entire genome, although this enrichment is significantly lower than that within enhancers (Figure R5A). Moreover, these raQTL scores significantly correlate with

∆𝑟𝑒𝑝𝑟𝑒𝑠𝑠𝑖𝑜𝑛 scores (𝑝 < 10^!%$^, Figure R5B), consistent with the findings among the CNS SNPs (Figure 7) and the SNPs in immune biosamples (Figure 8).

It is also noteworthy that the density of raQTLs in predicted silencers is significantly higher than that in other H3K27me3 ChIP-seq peaks (𝑝 < 10^!"$^, Figure R5A), supporting the active state of predicted silencers in comparison to other H3K27me3 ChIP-peaks again.

Figure R5 Evaluation based on raQTLs. (A) raQTLs are enriched in the predicted silencers and MPRA silencers as compared to the expected in the whole genome and non-predicted-silencer H3K27me3 ChIP-seq peaks (label as nonSL H3K27me3 in the figure). The asterisks and the number over the bars suggest the enrichment p value as compared to the whole genome. ∗∗

: 𝑝 < 10^!"#^. (B) ∆𝑟𝑒𝑝𝑟𝑒𝑠𝑠𝑖𝑜𝑛 scores significantly correlate with raQTL scores, regardless in predicted silencers or enhancers.

We summarized these results (Page 21) as follows.

“*To directly evaluate the relationship between* ∆𝑟𝑒𝑝𝑟𝑒𝑠𝑠𝑖𝑜𝑛 *scores and raSNPs, we resorted to the outcomes of MPRA experiments that assess allele-specific impacts of SNPs on gene regulation. Although these MPRA platforms were not specifically tailored for silencer SNPs, they provide valuable insights. For example, in SuRE MPRA experiments conducted in K562 cells (van Arensbergen et al. 2019), 19,237 SNPs were reported to significantly alter regulatory activity, known as reporter assay QLTs (raQTLs). These raQLTs are extremely enriched in K562 enhancers,*

*consistent with previous findings (van Arensbergen et al. 2019). Nevertheless, we also observed a significant enrichment of raQTLs in candidate silencers and K562 MPRA silencers compared to the whole genome and H3K27me3 ChIP-seq peaks not classified as silencers (binomial test* 𝑝 < 10^!"$^*), although these silencer enrichment levels are notably lower than that in enhancers (*𝑝 < 10^!"$^*, Figure S20A), as expected from the nature of the experimental data. This enrichment further supports the active state of K562 candidate silencers. In addition,* ∆𝑟𝑒𝑝𝑟𝑒𝑠𝑠𝑖𝑜𝑛*s are positively correlated with raQLT scores, irrespective of whether these raQTLs are in silencers or enhancers (Figure S20B). Taken together, MPRA scores by which the difference in regulatory influence between SNP alleles are quantified, though not specifically designed for silencer SNPs, can be used to examine the performance of* ∆𝑟𝑒𝑝𝑟𝑒𝑠𝑠𝑖𝑜𝑛*s in prioritizing disease-risk SNPs within candidate silencers.”*

5) To support the functional relevance of the study, the authors should provide experimental assessment of disease-associated silencers by performing CRISPR-mediated deletion or mutation of silencer candidates in relevant cell lines.

We appreciate the reviewer’s comment and have examined the experimental results to evaluate the activity of predicted silencers. Among the MPRA validated silencers in the biosample K562 (as detailed above), we observed that 541 MPRA silencers overlap with TREDNet silencers in K562. This number represents a significant enrichment as compared to the expected among DNase-seq peaks randomly selected from alternative biosamples and among K562 H3K27me3 ChIP-seq peaks having no overlap with the predicted silencers in K562 (𝑝 < 10^!"$^, Figure R6A). Similarly, we used the silencers detected by the ReSE MPRAs (Pang and Snyder 2020) to evaluate the activity of predicted silencers in HepG2 cells. It is evident that predicted silencers overlap with MPRA validated silencers significantly more often than expected by chance (𝑝 < 10^!"$^ as compared to the whole genome and other H3K27me3 ChIP-seq peaks, Figure R6B). These results further extend independent experimental validation of predicted silencers in two biosamples.

Figure R6 Significant overlap between predicted silencers and MPRA experimentally validated silencers in the biosamples

(A) K562 and (B) HepG2. ∗∗: 𝑝 < 10^!"#^. “nonSL H3K27me3” represents non-predicted-silencer H3K27me3 ChIP-seq peaks.

We added these results as follows (Page 7).

*“Additionally, we compiled 7,701 K562 silencers from two independent MPRA studies based on ReSU (Pang and Snyder 2020) and STARR-seq (Doni Jayavelu et al. 2020). Of them, 541 overlap with K562 predicted silencers, which represents a significant enrichment compared to the DNase-seq peaks randomly selected from alternative biosamples and H3K27me3 ChIP-seq peaks not predicted as silencers in K562 (binomial test* 𝑝 < 10^!"$^*, Figure S6A). Similarly, in HepG2, predicted silencers are*

*significantly enriched with silencers detected by the ReSU MPRA* (Pang and Snyder 2020) *(*𝑝 < 10^!"$^*, Figure S6B).”*

6) In addition to focusing on specific diseases, the full list of GWAS traits significantly enriched with silencers or enhancers should be provided.

We added the full list of GWAS traits significantly associated with enhancers or silencers in each tested biosample to the Table S3.

7) Figure 7C-D: the lead as well as the relevant LD-SNPs should be highlighted in panel C.

We modified Figure 7 to indicate the lead SNPs for Parkinson’s disease.

8) Introduction (Lines 53-56). When mentioning the recent advancements in massively parallel reporter assays (MPRAs) to map silencers genome-wide, additional references should be included: Doni Jayavelu et al. 2020, Hussain et al. 2023 and Xiusheng et al. 2023.

Thank you for the citations. We have added these references accordingly.

9) In general, the figures contain too much information and make sometime difficult to extract the relevant information. Simplification, moving some panels to supplemental figures or more specifically chosen examples to support the most important conclusions could be beneficial to the legibility.

We have simplified the figures. In Figure 1, we relocated C, D, F panels to the Supplementary Notes, as these plots do not pertain to the primary topics of this study. Additionally, we excluded the example rs62057068 from Figure 7, as we did not elaborate on this SNP in the text. Figures 8A, C, and D have been simplified by removing the results on “silencer-concordant-INSG” and “silencer- discordant-INSG”, as these results were not discussed in the text.

10) Line 71: "accuracy of silencer prediction was on par with our prior model". More information on the difference between the present and prior model could be beneficial to understanding the significance of the 0.84 AUC ROC, and why we should prefer a second model when it gives no improvement in AUC ROC compared to the prior model.

To demonstrate the improvement of TREDnet models in silencer prediction, we performed an independent testing on MPRA silencers, as detailed in the response to the Reviewer’s concern #2 and Figure R4A. These results showcase the advantage of the TREDnet over other models and the bare- bones H3K27me3 ChIP-seq signal, particularly in comparison to the SVM model and H3K27me3 signal, supporting our use of the TREDnet model.

Additionally, we have added the following lines to suggest the significancy of TREDnet models in the manuscript (Page 4).

*“… the accuracy of silencer prediction … was significantly better than AUROC = 0.77 of our prior SVM model (Huang et al. 2019). While the SVM model employs DNA sequences and gene expression profiles for silencer prediction, the TREDnet model is DNA sequence-based, and thus can be readily extended to additional biosamples.”*

11) Line 104-107: For the 3 loci mentioned, PCDH, HOXA and HOXD, with enrichment in

silencers, It would important to see a screenshot of these loci showing the predicted enhancer and silencers.

We have added the screenshots of predicted silencers and enhancers in immune biosamples as Figure R7 and referred to these figures accordingly as follows.

*“For example, the loci of PCDHA/G genes, which is highly conserved in vertebrates (Yu et al. 2007) and plays an important role in epithelial barrier formation and repair, displays the enrichment in candidate silencers, but not enhancers, in immune biosamples (Figure S4). The trend is also evident in the highly conserved loci of HOXA and HOXD clusters (Figure S4), …*” (Page 6).

Figure R7 Profiles of predicted silencer and enhancer across immune biosamples in (A) the loci of PCDH genes, (B) the loci of HOXA genes, (C) the loci of HOXD genes. All these gene loci are enriched with candidate silencers in immune biosamples.

12) Line 133/Figure 2E: figure legend is missing in the text. What does the dotted line mean, and how the number of 71.4% of CNS cells that are mentioned in the text (Line 133) to have dense silencer populations can be deduced? Is this percentage to be found in the figure?

We expanded the legend of Figure 2E specifying that the blue and yellow boxes represent silencers and enhancers, respectively. The dashed lines mark the threshold of significant enrichment

(1.9 × 10^!#^). We incorporated these details into the Figure 2E caption as follows.

*“The dash lines represent the threshold (*𝑝 = 1.9 × 10^!#^*) of significant enrichment (see Supplementary Notes).”*

The number of 71.4% mentioned in text can be deduced based on Figure 2E since the upper and lower whisker edges in a boxplot approximately represent the 25% and 75% quartiles of data distribution. We added the following line in the figure’s caption.

*“The upper and lower whisker edges in these boxplots approximately represent the 25% and 75% quartiles of the presented data.”*

Also to avoid a possible confusion (the number is not explicitly presented in Figure 2E), we deleted this reference to Figure 2E and revised this sentence as follows (Page 9).

*“The IRF4 locus, which is vital for the immune system, displays dense silencer populations in 71.4% of CNS cells.”*

Moreover, in the Supplementary Notes, we added a section to elaborate how we evaluate the enrichment of predicted silencers or enhances in each gene locus as follows.

*“Locus-specific enrichment of silencers and enhancers.*

*We used gene annotations from GENCODE (Frankish et al. 2021). The locus of a gene encompasses the gene body along with its two flanking upstream and downstream intergenic regions. Using this annotation, there are 26,550 gene loci in the human genome. For a given gene locus (say, g) and a biosample, the count of candidate silencers located within this locus was tallied, and the silencer enrichment significance was determined using the binomial test, i.e.,*

𝑝(𝑋 > 𝑛) = ∑^(^

8𝑁: 𝑝

';1 − 𝑝

=(!' , 𝑝

= ,$*,	(1)*

')*+" 𝑖	&	&	&	-

*where n and N are the numbers of silencers within the gene locus g and in the whole genome, respectively.* 𝑙_&_ *and L denote the length of the locus g and the whole genome, respectively. Using Bonferroni multiple-testing correction, the silencer density in the locus g is regarded as significantly*

*higher than expected in the whole genome when* 𝑝(𝑋 > 𝑛) ≤ 0.05/𝐺*. Here, G is the total number of*

*gene loci in the whole genome. Similarly, the significancy of enhancer enrichment in a gene locus is assessed based on enhancer counts.”*

Typos

line 43: isolators = insulators

Changed “isolators” to “insulators”.

Line 70: "building a multi-label classifier for each of biosamples collected …" should be "building a multi-label classifier for each of the biosamples collected …".

Corrected.

Line 105: "Display" should be "Displays"

Corrected.

Line 112: "although not comprehensively enough" should be "although not comprehensive enough".

Corrected.

Line 133: "display" should be "displays"

Corrected.

Line 191: "2.2 million GWAS SNPs were complied" should be "2.2 million GWAS SNPs were compiled"

Corrected.

Line 227: "underlaying" should be "underlying"

Corrected.

line 245: nature killer = natural killer

Corrected.

line 294: "pervious"= should be "previous"

Corrected.

line 360: provide year of publication for reference "Cooper et al."

We revised this citation, adding the publication year.

line 462: "the disruption GABAergic interneuron » should be "the disruption of the GABAergic interneuronS"

Corrected.

line 452: "majority" should be "the majority"

Corrected.

line 452: "are DFREs" should be "as DFREs"

Corrected.

line 453: "which still can be elevated" should be "which could still increase"

Corrected.

*line 216: The "locus TLR9" is not displayed in Figure 4C.*

We TLR9 to Figure 4C. TLR9 is represented by the rightmost column in this plot now.

*Figure 4D: Asterixis missing to denote significance mentioned in line 242?*

We added asterisks in Figure 4D.

*Figure 6D: TF gene names are illegible.*

We used the “Approved symbol” from https://[www.genenames.org/](http://www.genenames.org/) for each TF gene listed in this figure and Figure S18 and revised the font size to make gene names legible.

Reviewer #2: Huang et al. presented an interesting study that explored the potential functions of genetic variants in non-coding silencer elements. By first making a deep learning prediction on silencers and enhancers, they further studied the enrichment of GWAS SNPs in these two non- coding elements. They identified a few examples indicating the causal effects of SNPs within silencers in different diseases. The study is sound, and the knowledge gained is important, as silencers have not been studied broadly, and most of the time, only enhancers were considered and studied when interpreting genetic variants in the human population.

We are very grateful to this Reviewer for the positive and very supportive opinion of the presented results. We wholeheartedly agree that the research of silencers has been incomparably low scale in comparison to the enhancer research field. We hope that the presented results of abundant disease-associated mutations in candidate silencers could serve as a catalysator of silencer-centered research.

There are some points that may improve the manuscript.

1. How many predicted silencers overlap with the experimentally identified silencers?

This is a very important concern residing at the foundation of our study, which has similarly been raised by the first Reviewer. To extend independent experimental validation of TREDNet silencer predictions, we assembled several additional experimental silencer datasets and performed additional validation analyses. In a brief summary of our corresponding response to the Reviewer #1, we collected silencers experimentally identified using silencer-specific MPRA platforms in K562 and HepG2 cells. In K562, from two studies ReSE (Pang and Snyder 2020) and STARR-seq (Doni Jayavelu et al. 2020), we gathered 7,701 MPRA silencers. Of these silencers, 542 MPRA silencers overlap with predicted silencers in K562. This number represents a significant enrichment as compared to the expected by chance using DNase-seq peaks randomly selected from alternative biosamples and K562 H3K27me3 ChIP-seq peaks not overlapping with predicted silencers in K562 (𝑝 < 10^!"$^, Figure R8A). Similarly, in the HepG2 cells, predicted silencers significantly overlap with the silencers experimentally identified using ReSE MPRAs (Pang and Snyder 2020)( 𝑝 < 10^!"$^, Figure R8B). These enrichments support the negative and active regulatory impact of predicted silencers.

Furthermore, we also used the MPRA scores from other two studies--the Sharpr-MPRA (Ernst et al. 2016) and ATAC-STARR-seq (Hansen and Hodges 2022). Both studies developed an MPRA platform to measure the silencing and activating effect of genomic sequences. In the K562 and HepG2 cells, predicted silencers frequently display negative Sharpr-MPRA scores. These scores are significantly lower than those observed in enhancers and all regions tested (Figure R8C). Similarly, in GM12878 cells, there is a notable enrichment of significantly negative ATAC-STARR-seq scores among predicted silencers compared to all tested DNA sequences and enhancers, supporting their active silencing effect (Figure R8C).

Finally, we examined the TREDnet model on MPRA silencers. Among 7,701 MPRA silencers, 6,999 have no overlap with training sequences in K562. On these 6,999 MPRA silencers, TREDnet has the

classification performance with AUROC = 0.74 and AUPRC = 0.30. These values suggest that TREDnet performs slightly better than our prior CNN model (AUROC = 0.75 / AUPRC = 0.24) and significantly outperforms our prior SVM model (AUROC = 0.63 / AUPRC = 0.18) as well as the H3K27me3 profiles (AUROC = 0.58 / AUPRC = 0.16, Figure R8C). These comparisons reaffirm the effectiveness of TREDnet in predicting active silencers, in K562 in particular.

Figure R8. Evaluation of candidate silencers and TREDnet models. Overlap between MPRA silencers and candidate silencers in (A) K562 and (B) HepG2 cells. Candidate silencers are compared with H3K27me3 peaks lacking candidate silencers and background elements (DNase-seq peaks randomly selected from alternative biosamples). Asterisks on the top of bars point to significant overlap as compared to the background; ∗∗ 𝑝 < 10^!"#^. (C) MPRA scores of predicted silencers. (D) classification performance of MPRA silencers.

We added these results to the manuscript as described in our response to the Reviewer #1.

2. It would be interesting to integrate the eQTL data to check if the genetic changes in silencers would affect the nearby genes.

To address this, we downloaded eQTL data from the GTEx project (The GTEx Consortium 2015) for 17 distinct tissues, comprising 13 brain tissues, colon, lung, spleen, and whole blood. For each GTEx tissue, we checked the distribution of eQTLs within candidate silencers in the corresponding biosamples. For example, we gathered eQTLs from all brain GTEx tissues and examined their density within candidate silencers in each brain biosample. In the end, 40 biosamples were tested in this analysis.

We found that candidate silencers exhibit significant enrichment of eQTLs compared to the whole genome in the majority of tested biosamples (28 out of 40, Figure R9A). Additionally, silencer eQTLs show comparable significance levels as enhancer eQTLs across all tested biosamples (Figure R9B). In addition, in 90% of examined biosamples (36/40), silencer eQTLs show a significantly higher tissue specificity than enhancer eQTLs (Figure R9C).

Figure R9 EQTLs in candidate silencers across biosamples. (A) Densities, (B) eQTL slopes and (C) tissue-specificities of eQTLs within candidate silencers and enhancers. Asterisks in (A) represent the significant enrichment as compared to the whole genome (p<0.05). Asterisks in (C) represent the significant tissue-specificity levels of candidate silencer eQTLs as compared to enhancer eQTLs. For a given eQTL, a low number of tissues in which this eQTL was detected suggests a high tissue specificity of this eQTL.

We included these results into the revised manuscript (Page 13).

*“…, candidate silencers exhibit significant enrichment in expression quantitative trait loci (eQTLs) obtained from the GTEx project (The GTEx Consortium 2015) compared to the whole genome across 28 out of 40 examined biosamples (Figure S12A, see Supplementary Notes). Additionally, candidate silencer eQTLs achieve significance levels akin to enhancer eQTLs across these biosamples (Figure S12B). Silencer eQTLs are, however, more tissue-specific than enhancer eQTLs in 90% of examined biosamples (36/40;* 𝑝 < 0.05*, Figure S12C). ……Taken together, these findings suggest an observable phenotypic impact of candidate silencers.”*

3. Have the authors also compared their analysis with the ClinVar data to see any more diseases related variants fall within silencers? Or other cancer related database with disease-associated mutations.

As suggested by the Reviewer, we extended our analysis to the ClinVar data. Specifically, we examined the density of GWAS SNPs cataloged by the ClinVar (Landrum et al. 2020) and noted that predicted silencers host 1.47 ClinVar SNPs per 1kbp. This number exceeds 1.29 ClinVar SNPs per 1kbp found within enhancers, both significantly surpassing the expected 0.76 per 1kb from the whole genome baseline (𝑝 < 10^!"$^*,* Figure R10A). Additionally, we explored cancer somatic variants compiled in the ICGC database (Zhang et al. 2011). These somatic variants show enrichment within candidate silencers in matched biosamples (Figure R10B). These findings, together with those based on GTEx eQTLs and NHGRI GWAS SNPs, suggest a significant phenotypic contribution of candidate silencers.

Figure R10 Enrichment of ClinVar SNPs and cancer somatic variants in candidate silencers across biosamples. (A) The density of ClinVar SNPs in candidate silencers and enhancers across all tested biosamples. The dash line represents the density of ClinVar SNPs in the whole genome. Asterisks above boxplots represent a significant enrichment as compared to the whole genome; ∗∗ 𝑝 < 10^!%^. (B) The density of cancer somatic variants within candidate silencers in matched biosamples. The blue and orange asterisks above bars represent a significant within candidate silencers and enhancers enrichment compared to the whole genome (p<0.05), respectively.

We summarized these results in the manuscript (Page 13).

“*Furthermore, we explored the distribution of GWAS SNPs deposited to the ClinVar archive (Landrum et al. 2020). Candidate silencers host 1.47 ClinVar SNPs per 1 kb. This density exceeds 1.29 ClinVar SNP per 1 kb within enhancers, with both densities significantly surpassing the expected 0.76 ClinVar SNP per 1 kb baseline from the whole genome (*𝑝 < 10^!.^, *Figure S13A). We also examined the distribution of cancer somatic variants compiled in the ICGC database (Zhang et al. 2011). These cancer variants show significant enrichment within candidate silencers in the matched biosamples for seven out of eight examined cancers (Figure S13B). For example, the density of myeloid variants in K562 candidate silencers is 1.3 times that expected from the whole genome baseline.”*

4. The authors seem to use SNP and mutations interchangeably. Would be better to clarify the definition of these two events.

To avoid any ambiguity and clarify the meaning of the reported variants largely specific to the natural variation within the human population, we have resorted to the use of the “variant” term unless a

specific SNP or SNP dataset is referenced. In particular, we changed the title of this manuscript from “The contribution of silencer mutations to human diseases” as “*The contribution of silencer variants to human diseases*”.

For example:

*“…, as predicted by SNP-SELEX deltaSVM (Yan et al. 2021), the variant at this SNP gains a binding site for NFE2, …” (Page 23)*

*“TF-motif-mapping analysis also shows increased binding affinity of FOXC2 due to the G to A change at this SNP (Figure 7E).” (Page 23)*

*“…the binding motif mapping analysis suggests that this SNP variant potentially disrupts a binding site for NFKB1,…” (Page 25)*

5. The manuscript will benefit from better editing of the figures and more detailed figure legends.

We streamlined the figures and revised figure legends. In Figure 1, we relocated C, D, F panels to the supplementary notes as these plots do not pertain to the primary topics of this study. Additionally, we omitted the example rs62057068 in Figure 7, as we did not elaborate this SNP in the text. Figures 8A, C, and D have been simplified by excluding the results of “silencer-concordant-INSG” and “silencer- discordant-INSG”, since these results were not discussed in the main text.

Minor

Line 43: isolators – insulators

Corrected.

Fig. 1C. It is complicated to have 3 subgraphs in 1 panel, which is hard to understand the data.

For simplification, we added labels to all axes in Fig. 1C (which was moved to Supp Materials as Fig. S1 to simplify Figure 1).

Fig. 2A. There are no units on the y-axis.

We added tick labels to y-axes in Figure 2A.

Fig. 7. A few motifs related to SNPs have no explanation in the legend.

We removed the results pertaining to the SNP rs62057068 since we did not discuss this SNP in the text.

Doni Jayavelu N, Jajodia A, Mishra A, Hawkins RD. 2020. Candidate silencer elements for the human and mouse genomes. *Nature Communications* **11**: 1061.

Ernst J, Melnikov A, Zhang X, Wang L, Rogov P, Mikkelsen TS, Kellis M. 2016. Genome-scale high-resolution mapping of activating and repressive nucleotides in regulatory regions. *Nature Biotechnology* **34**: 1180.

Frankish A, Diekhans M, Jungreis I, Lagarde J, Loveland Jane E, Mudge JM, Sisu C, Wright JC, Armstrong J, Barnes I et al. 2021. GENCODE 2021. *Nucleic Acids Research* **49**:

D916-D923.

Hansen TJ, Hodges E. 2022. ATAC-STARR-seq reveals transcription factor-bound activators and silencers across the chromatin accessible human genome. *Genome Research* doi:10.1101/gr.276766.122.

Huang D, Ovcharenko I. 2022. Enhancer–silencer transitions in the human genome.

*Genome Research* **32**: 437-448.

Huang D, Petrykowska HM, Miller BF, Elnitski L, Ovcharenko I. 2019. Identiﬁcation of human silencers by correlating cross-tissue epigenetic proﬁles and gene expression.

*Genome Research*.

Hudaiberdiev S, Taylor DL, Song W, Narisu N, Bhuiyan RM, Taylor HJ, Tang X, Yan T, Swift AJ, Bonnycastle LL et al. 2023. Modeling islet enhancers using deep learning identiﬁes candidate causal variants at loci associated with T2D and glycemic traits. *Proc Natl Acad Sci U S A* **120**: e2206612120.

Landrum MJ, Chitipiralla S, Brown GR, Chen C, Gu B, Hart J, Hobman D, Jang W, Kaur K, Liu C et al. 2020. ClinVar: improvements to accessing data. *Nucleic Acids Research* **48**: D835-D844.

Pang B, Snyder MP. 2020. Systematic identiﬁcation of silencers in human cells. *Nature Genetics* **52**: 254-263.

Salameh TJ, Wang X, Song F, Zhang B, Wright SM, Khunsriraksakul C, Ruan Y, Yue F. 2020. A supervised learning framework for chromatin loop detection in genome-wide contact maps. *Nature Communications* **11**: 3428.

The GTEx Consortium. 2015. The Genotype-Tissue Expression (GTEx) pilot analysis: Multitissue gene regulation in humans. *Science* **348**: 648-660.

van Arensbergen J, Pagie L, FitzPatrick VD, de Haas M, Baltissen MP, Comoglio F, van der Weide RH, Teunissen H, Võsa U, Franke L et al. 2019. High-throughput identiﬁcation of human SNPs abecting regulatory element activity. *Nature Genetics* **51**: 1160- 1169.

Yan J, Qiu Y, Ribeiro Dos Santos AM, Yin Y, Li YE, Vinckier N, Nariai N, Benaglio P, Raman A, Li X et al. 2021. Systematic analysis of binding of transcription factors to noncoding variants. *Nature* **591**: 147-151.

Yu WP, Yew K, Rajasegaran V, Venkatesh B. 2007. Sequencing and comparative analysis of fugu protocadherin clusters reveal diversity of protocadherin genes among teleosts. *BMC Evol Biol* **7**: 49.

Zhang J, Baran J, Cros A, Guberman JM, Haider S, Hsu J, Liang Y, Rivkin E, Wang J, Whitty B et al. 2011. International Cancer Genome Consortium Data Portal--a one-stop shop for cancer genomics data. *Database : the journal of biological databases and curation* **2011**: bar026-bar026.

**Second round of review**

**Reviewer 1**

The authors have addressed most of my remarks and the manuscript has been substantially improved. However, my remark #5 regarding functional validation by CRISPR was not addressed. Although I understand that it might be time consuming to perform such experiments, this issue should be at least mentioned in the discussion to acknowledge the fact that functional validation will be needed to assess the physiological relevance of identified silencer-associated variants.

In addition, several typos should be corrected:
raQTLs are written raQLT in several instances.

“since genes associated with active silencers are likely to be lowly expression” should be: “since genes associated with active silencers are likely to be lowly expressed”

“consistently display notably low expression across all tested biosamples” should be: “consistently display low expression across all tested biosamples”

**Reviewer 2**

The authors have addressed my concerns properly.

**Authors’ response to reviewers**

- We added the following statement about the CRISPR test on silencers to address the comment of Reviewer #2: “Furthermore, MPRA results and gene expression analysis have shown that candidate silencers suppress gene transcription levels in several biosamples. Additional experimental examinations on candidate silencers across more biosamples and using different platforms (such as CRISPR) will be beneficial for future silencer investigations.” (Page 28)
- We corrected the typos: “raQLT” to “raQTL”, “lowly expression” to “lowly expressed”, “consistently display notably low expression” to “consistently display low expression”.
